# *N*,*N*-Dimethylformamide’s Participation in Domino Reactions for the Synthesis of Se-Phenyl Dimethylcarbamoselenoate Derivatives

**DOI:** 10.3390/molecules30030747

**Published:** 2025-02-06

**Authors:** Runsheng Xu, Shenhuanran Hu, Luhui Wu, Yifan Ning, Jin Xu

**Affiliations:** College of Life and Health, Huzhou College, Huzhou 211300, China; com1241613607@163.com (S.H.); wuluhui141@gmail.com (L.W.); fansxmy@outlook.com (Y.N.)

**Keywords:** dual role, DMF, domino reaction, C–selenium bond formation, copper catalyst

## Abstract

*N*,*N*-dimethylformamide’s (DMF) participation in domino reactions has been developed. Starting from substituted halogenobenzenes and selenium powder, versatile biologically active Se-phenyl dimethylcarbamoselenoate derivatives were efficiently synthesized under mild reaction conditions. The reaction mechanism was studied using control experiments. These protocols involve a wider substrate scope and provide an economical approach toward C–selenium bond formation.

## 1. Introduction

**Scheme 1:**
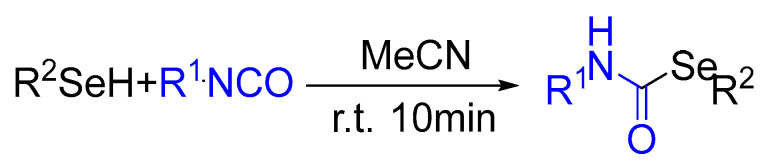
Click reaction of selenols with isocyanates.

Selenium compounds are widely used in various fields, such as synthetic chemistry, pharmaceutical pesticides, and functional materials [1,2]. Furthermore, selenium is one of the most important markers in the human body and has a variety of important functions for human health. First, selenium can enhance the ability of the immune system to identify pathogens in the body. Selenium also helps to maintain the sensitivity of the nervous system [3,4]. Secondly, selenium is an effective antioxidant that can eliminate free radicals in the body, reducing oxidative damage to cells and thereby preventing and reducing the severity of certain chronic diseases. Selenium can reduce cholesterol levels in the blood, prevent the occurrence of arteriosclerosis, inhibit platelet agglomeration, and preserve cardiovascular health [5,6,7]. Selenium also has a positive impact on male reproductive health, improving sperm quality and enhancing fertility. In terms of the digestive system, selenium can improve and enhance the absorption function of the digestive system, accelerate gastrointestinal motility, promote the decomposition and absorption of gastrointestinal content, and help to reduce the symptoms of indigestion [8,9,10,11]. Taninia reported a click reaction of selenols with isocyanates (Scheme 1) [12]. In this method, selenols react with isocyanates under mild catalyst-free conditions to generate selenocarbamates in good yield and with high selectivity over potentially competing nucleophilic additions. The methodology enables the incorporation of a wide variety of functional groups, providing access to a broad array of densely functionalized selenocarbamates. Therefore, the synthesis of organic selenium compounds with a variety of functions is of great significance.

Being a favorable solvent, *N*,*N*-dimethylformamide (DMF) is also an important synthetic structure [13,14,15]. It can be used as a parental reagent, a nuclear test agent, and in free radical reactions. It can participate in multiple chemical reactions in organic synthesis. For example, DMF plays an important role in methyl-based reactions. Through the Vilsmeier reagent, the metacimal part of DMF is transferred to the double bond to realize alfalized compounds, heterogenic circular compounds, and pitro-enriched reactions of the e-electronic olefin. DMF can also react with metal reagents to produce aldehydes [16]. When bromine aromatics or iodine-aged aromatics respond to metal halogen with organic lithium reagents or grid reagents, the generated aromatherapy reagent reacts with DMF to efficiently prepare a series of aromatic Aldo [17]. DMF also plays an important role in a vital method for reducing the amino reactor aldosone to amine. For example, a Lewis acid catalyzes the DMF triangle to participate in restorative amino reactions [18]. The reaction substrate is very broad, and the functional group is also very inclusive. In cyanide reactions, DMF can also be used as a C-H functionalized reagent [19,20,21].

In addition to the above reactions, DMF can be used as a ligand that participates in metal catalytic reactions to achieve amino-based reactions of halogen aromatics. Furthermore, DMF can be used as an important source of dihylaminel, and it can be utilized to realize bigramine-based reactions of various marinated hydrocarbons. Moreover, DMF can participate in the reaction of a cord additional [2+2] bonus to a cyclolar bonus. In summary, DMF plays multiple roles in organic synthesis and can participate in multiple chemical reactions. Its unique structure and properties make DMF a very useful tool in synthesis, providing new means and methods for organic synthesis.

Our interest focuses on traditional metal-catalyzed C-H bond functionalization. Over the past decade, many reactions involving S-C and Se-C bond formation have been developed by our group [22,23,24]. Herein, a dual role of DMF’s participation in domino reactions has been developed. Starting from substituted halogenobenzenes and selenium powder, versatile biologically active Se-phenyl dimethylcarbamoselenoate derivatives were efficiently synthesized under mild reaction conditions. The reaction mechanism was studied by means of deuterium isotope experiments. These protocols involve a wider substrate scope and provide an economical approach toward C–selenium bond formation.

## 2. Results and Discussion

At the beginning of our experiments, we investigated the model reaction of iodobenzene **1a** and selenium powder **2** to study the reaction conditions, including the optimization of catalysts, bases, and solvents. As shown in Table 1, at the outset, copper salts were used as the catalyst (entries 1–6), and no desired product was gained when the reaction was conducted in the presence of CuO as the catalyst in DMSO (entry 1). These results show that using the proper solvent is critical for this reaction as, when the reactions were conducted in an apolar solvent, the DMF product was detected in a moderate yield. CuBr_2_ was proven to be the most efficient catalyst species in this reaction (entry 5). Gratifyingly, the yield of product **4a** was obtained at 75% when the catalyst was changed to Cu(OAc)_2_ (entry 6). By screening different bases for the reaction, Cs_2_CO_3_ was demonstrated to be a more suitable base than others such as NaOH, Na_2_CO_3_, Na_2_SO_4_, NaOEt, K_2_CO_3_, and K_2_PO_3_ (entries 6–12). A reduced yield was obtained in the reactions operated at 100 °C (72% yield, entry 13) and 120 °C (77% yield, entry 13). We also found that the yields of the product decreased when the amount of copper catalyst used was higher or lower than 10 mol% equivalent (entries 17 and 18). Finally, we determined that the optimal reaction conditions were as follows: Cu(OAc)_2_ used as the catalyst, Cs_2_CO_3_ used as the base, a ratio of **1a**:**2** of 1:1.5:1, a N_2_ atmosphere, 110 °C, and preparation for 24 h.

Next, the substrate scope was examined under the optimal conditions, and the results are shown in Table 2. Aryl iodides **1,** selenium powder **2,** and DMF **3** were subjected to this reaction and the products were produced in good to excellent yields (79–92%). A variety of functional groups, including methyl, methoxy, halogen, and naphthyl groups, were compatible with aryl iodides **1**. It was found that both the electron-donating and electron-withdrawing aryl iodides **1** reacted smoothly with selenium powder **2** and DMF **3**. Aryl iodides **1** bearing electron-withdrawing groups showed better activity than those bearing electron-donating groups. This suggests that the conjugated structure could strongly coordinate with the copper catalyst, providing good yields (**4g**, 92% yield; **4h**, 88% yield). Despite the strong electron-donating effect of the trimethyl group, the corresponding product **4f** was still obtained at a 79% yield.

After the group tolerance of aryl iodides **1** was demonstrated, the diversity of bromine-substituted benzene derivative **5** partners was further investigated under the optimized reaction conditions. A wide array of bromine-substituted benzene derivatives **5** were subjected to this reaction and the products were produced in moderate to good yields (70–86%). A variety of functional groups, including methyl, methoxy, halogen, and biphenyl groups, were compatible. The results are shown in Table 3. We also attempted to use strong electron-withdrawing groups such as trifluoromethyl and nitro under the current reaction conditions; however, this only led to the decomposition of the starting material without the expected product.

**Scheme 2:**
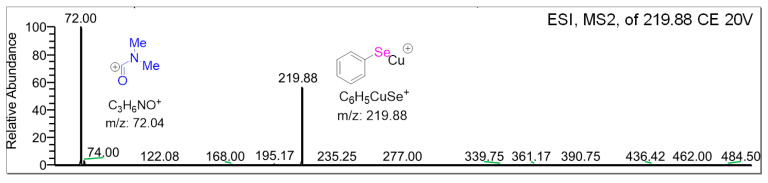
MS–MS analysis of the isolated product reaction mixture.

To gain a better understanding of this side reaction, tandem mass spectrometry (MS/MS) confirmed the position of the kinetic deuterium isotope effects, as shown in Scheme 2. In the mass spectrometry analysis, the fragment peak of *m*/*z* 219.88 (C_6_H_5_SeCu^+^) was absent. This demonstrates that oxidative addition is the rate-determining step for C-Se coupling in this reaction. Furthermore, the absence of the fragment peak of *m*/*z* 72.04 in the mass spectrometry analysis demonstrates that C_3_H_6_NO^+^ may be a key intermediate. 

**Scheme 3:**
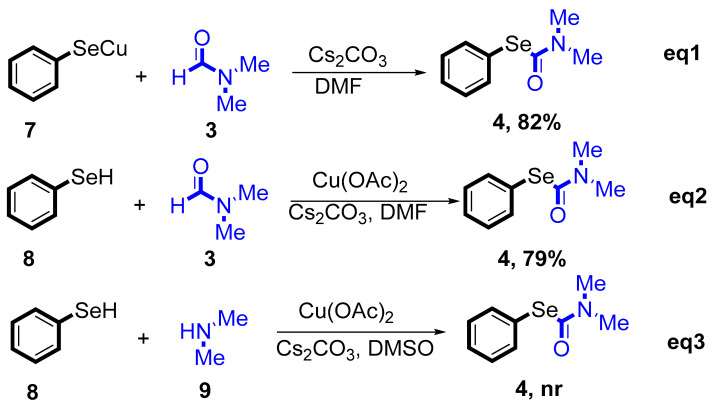
Preliminary mechanism investigation.

To gain more insights into the reaction mechanism, some selective and control experiments were performed (Scheme 3). We examined the chemical competence of PhSeCu under optimal conditions in the presence of benzothiazole under a N_2_ atmosphere, and the desired product **4a** was obtained in an 82% isolated yield (Scheme 3, eq1). These data for stoichiometric reactions of PhSeCu suggest that elemental selenium plays a key role in the process of C-Se formation, as shown in Scheme 3, eq2. This is consistent with our hypothesis that PhSeH may be a chemically competent intermediate, primarily through Ullman-type selenation between aryl iodides and selenium in situ during the catalytic cycle. Finally, through the addition of dimethylamine under the optimized reaction conditions (Scheme 3, eq3), the desired transformation was achieved. 

**Scheme 4:**
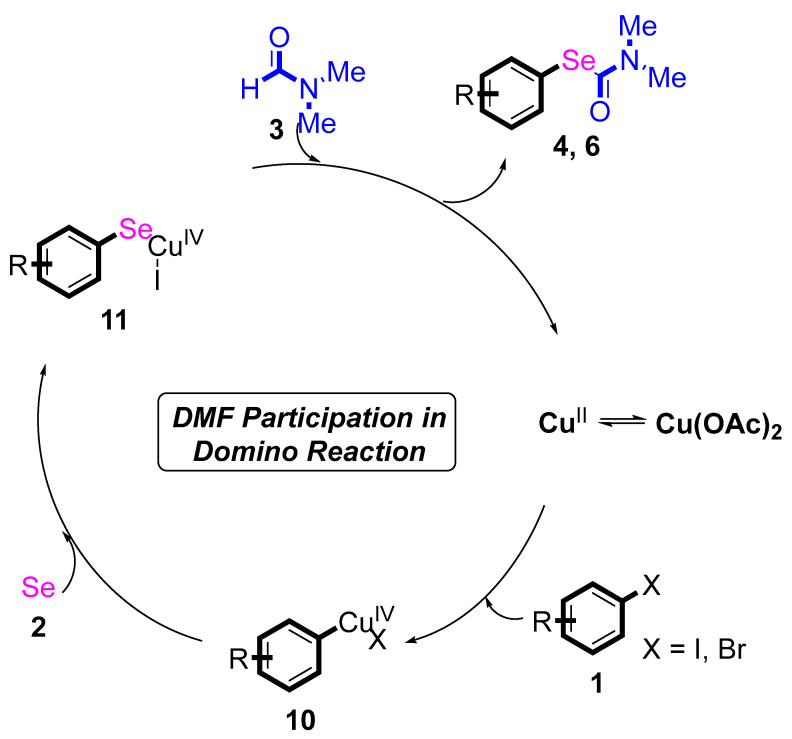
Proposed mechanism for N, N-dimethylformamide’s participation in domino reactions.

Based on the above results, a possible reaction mechanism is proposed (Scheme 4) [25,26,27,28]. At the beginning, the coordination process of Cu^II^ and substituted halogenobenzenes **1** generated a Cu^IV^ intermediate **10**. Then, the substituted halogenobenzenes **1** were converted to intermediate **11c** by reacting with the selenium powder. Finally, the desired products **4** and **6** were obtained via C–Se bond cross coupling from intermediate **11** with DMF. Then, the Cu^II^ species was generated, which re-entered the catalytic cycle.

## 3. Materials and Methods

All reagents used in experiment were obtained from commercial sources and used without further purification. Solvents for chromatography were technical grade and distilled prior for using. Solvent mixtures were understood as volume/volume. Chemical yields refer to pure isolated substances. Catalysts were purchased for analytical reagent. Thin layer chromatography employed glass 0.25 mm silica gel plates with F_254_ indicator, visualized by irradiation with UV light. Reactions were carried out under argon in flame-dried or oven-dried glassware unless otherwise specified. Dichloroethane, dichloromethane, acetonitrile, toluene (after distilling from sodium), dimethyl sulfoxide, and tetrahydrofuran (after distilling from sodium) were dried from 4Å molecular sieves. Synthesis-grade solvents were used after as purchased. Chromatographic purification of products was accomplished using silica gel (300–400 mesh). For thin layer chromatography (TLC) analysis, Merck pre-coated TLC plates (silica gel 60 GF_254_, 0.25 mm) were employed, using UV light as the visualizing agent. The compounds were isolated using Biotage flash column chromatography.

A mixture of iodobenzene **1a** (2.04 g, 10 mmol) and selenium powder **2** (0.79 g, 15 mmol), Cu(OAc)_2_ (182 mg, 10 mol%), Cs_2_CO_3_ (6.52 g, 2 equiv), DMF (10 mL). The tube was evacuated and refilled with N_2_ three times. The reaction is carried out under nitrogen protection. The reaction mixture was stirred at 110 °C for 12 h. After it was cooled, the reaction mixture was diluted with 20 mL of ethyl ether for 3 times. The filtrate was washed with water (3 × 15 mL). The organic phase was dried over Na_2_SO_4_, filtered, and concentrated under reduced pressure. and filtered through a pad of silica gel, followed by washing the pad of silica gel with the same solvent (20 mL). The residue was then purified by flash chromatography on silica gel to provide the corresponding product. The pure product Se-phenyl dimethylcarbamoselenoate **4a** was obtained 1.96 g, 86% yield. More experimental details can be found in the Supplementary Materials.

## 4. Conclusions

In summary, a dual role of *N,N*-dimethylformamide participation in domino reactions has been developed. Starting from substituted halogenobenzenes and selenium powder, versatile biologically active Se-phenyl dimethylcarbamoselenoate derivatives were efficiently synthesized under mild reaction conditions. The reaction mechanism was studied by means of deuterium isotope experiments. These protocols involve a wider substrate scope and provide an economical approach toward C–selenium bond formation.

## Figures and Tables

**Table 1:** Optimization of the reaction conditions *^a^*.

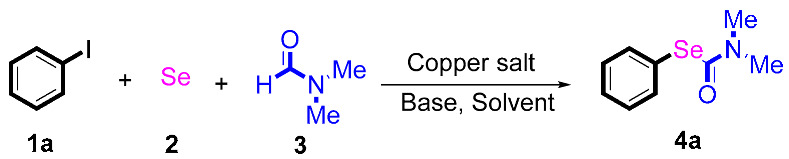					
Entry	Copper Salt	Base	Solvent	1a:2	Yield (%) *^b^*
1	CuO	Na_2_CO_3_	DMSO	1:1	0
2	CuSO_4_	Na_2_CO_3_	DMF	1:1	18
3	CuI	Na_2_CO_3_	DMF	1:1	29
4	CuCl_2_	Na_2_CO_3_	DMF	1:1	34
5	CuBr_2_	Na_2_CO_3_	DMF	1:1	60
6	Cu(OAc)_2_	Na_2_CO_3_	DMF	1:1	75
7	Cu(OAc)_2_	Cs_2_CO_3_	DMF	1:1	86
8	Cu(OAc)_2_	NaOH	DMF	1:1	56
9	Cu(OAc)_2_	Na_2_SO_4_	DMF	1:1	49
10	Cu(OAc)_2_	NaOEt	DMF	1:1	65
11	Cu(OAc)_2_	K_2_CO_3_	DMF	1:1	55
12	Cu(OAc)_2_	K_2_PO_3_	DMF	1:1	57
13	Cu(OAc)_2_	Cs_2_CO_3_	DMF	1:1	5
14	Cu(OAc)_2_	Cs_2_CO_3_	DMF	1:1	48
15	Cu(OAc)_2_	Cs_2_CO_3_	DMF	1:1.5	72 *^c^*
16	Cu(OAc)_2_	Cs_2_CO_3_	DMF	1:1.5	77 *^d^*
17	Cu(OAc)_2_	Cs_2_CO_3_	DMF	1:1.5	64 *^e^*
18	Cu(OAc)_2_	Cs_2_CO_3_	DMF	1:1.5	69 *^f^*

*^a^* Unless otherwise noted, reactions conditions were **1a** (10 mmol), **2** (10 mmol), copper catalyst (10 mol%), base (2 equivalent, under N_2_ atmosphere), solvent (10 mL), and 110 °C for 24 h. *^b^* Isolated yield. *^c^* 100 °C. *^d^* 120 °C. *^e^* Cu(OAc)_2_ (15 mol%). *^f^* Cu(OAc)_2_ (5 mol%).

**Table 2:** *N,N*-dimethylformamide’s participation in domino reactions *^a^*.

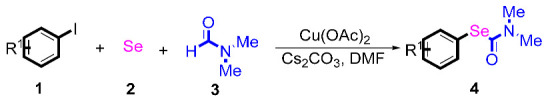			
Entry	1	Product	Yield (%) *^b^*
1	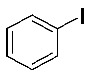	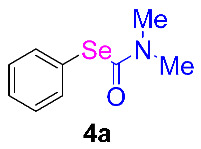	86
2	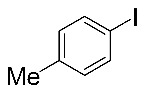	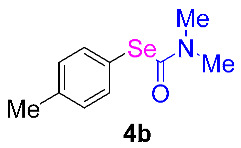	84
3	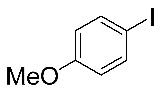	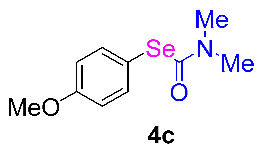	81
4	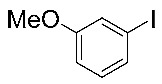	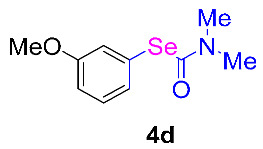	83
5	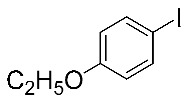	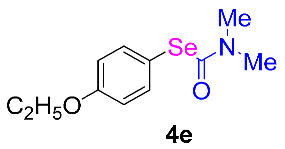	80
6	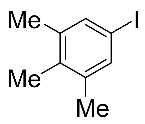	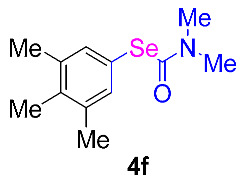	79
7	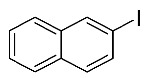	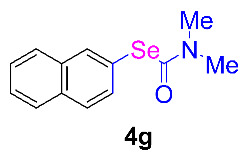	92
8	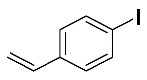	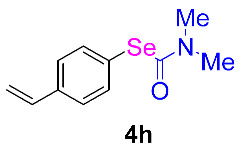	90
9	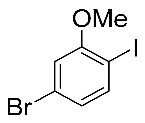	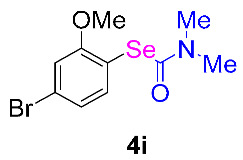	88
10	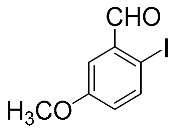	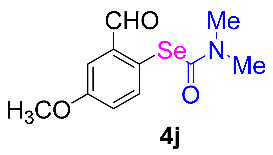	89

*^a^* Unless otherwise noted, the reaction conditions were as follows: **1** (10 mmol), **2** (15 mmol), Cu(OAc)_2_ (10 mol%), Cs_2_CO_3_ (2 equivalent), a N_2_ atmosphere, DMF (10 mL), and 110 °C for 24 h. *^b^* Isolated yield.

**Table 3:** *N*,*N*-dimethylformamide’ participation in domino reactions *^a^*.

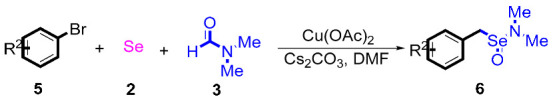			
Entry	5	Product	Yield (%) *^b^*
1	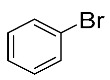	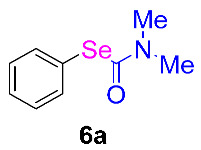	81
2	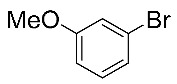	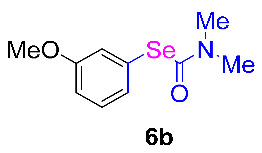	78
3	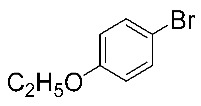	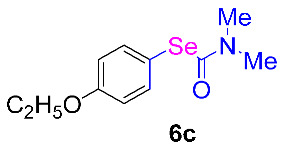	76
4	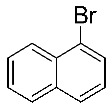	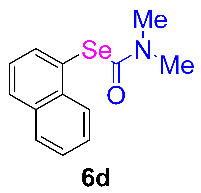	85
6	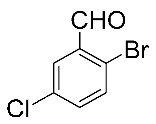	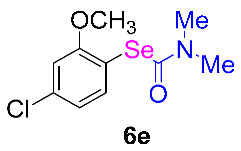	86
5	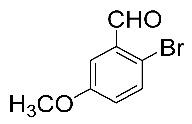	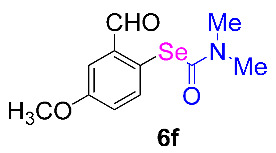	83
7	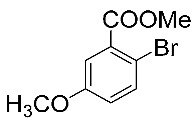	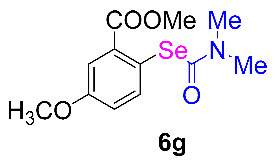	72
8	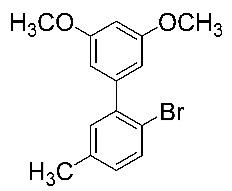	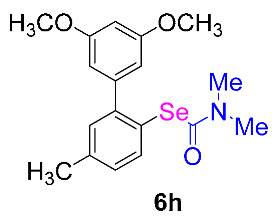	70

*^a^* Unless otherwise noted, the reaction conditions were as follows: **5** (10 mmol), **2** (15 mmol), Cu(OAc)_2_ (10 mol%), Cs_2_CO_3_ (2 equivalent), a N_2_ atmosphere, DMF (10 mL), and 110 °C for 12 h. *^b^* Isolated yield.

## Data Availability

All relevant data are within the paper.

## Supplementary Materials

The following supporting information can be downloaded at: https://www.mdpi.com/article/10.3390/molecules30030747/s1.
