# Supplementary material for: N,N-Dimethylformamide’s Participation in Domino Reactions for the Synthesis of Se-Phenyl Dimethylcarbamoselenoate Derivatives
Source: Molecules. 2025 Feb 6;30(3):747. doi: 10.3390/molecules30030747 (PMC11820005; doi:10.3390/molecules30030747)
Supplement: Supplementary file 1 [file molecules-30-00747-s001.zip › molecules-3436014-supplementary.pdf]

Supporting Information

Contents

General Information......S2

Optimization of the Reaction Condition......S3

General Procedures for Preparation of 4 and 6 ......S5

Preliminary Mechanism Investigation......S6

Analytical Data......S8

Spectrum......S15

General Information

All reagents used in experiment were obtained from commercial sources and used without further purification. Solvents for chromatography were technical grade and distilled prior for using. Solvent mixtures were understood as volume/volume. Chemical yields refer to pure isolated substances. Catalysts were purchased for analytical reagent. Thin layer chromatography employed glass 0.25 mm silica gel plates with F_{254} indicator, visualized by irradiation with UV light. Reactions were carried out under argon in flame-dried or oven-dried glassware unless otherwise specified. Dichloroethane, dichloromethane,

acetonitrile, toluene (after distilling from sodium), dimethyl sulfoxide, and tetrahydrofuran (after distilling from sodium) were dried from 4Å molecular sieves. Synthesis-grade solvents were used after as purchased. Chromatographic purification of products was accomplished using silica gel (300-400 mesh). For thin layer chromatography (TLC) analysis, Merck pre-coated TLC plates (silica gel 60 GF_{254}, 0.25 mm) were employed, using UV light as the visualizing agent. The compounds were isolated using Biotage flash column chromatography.

The NMR spectra were recorded at 400 MHz for ^{1}H, 100 MHz for ^{13}C,. The NMRs were recorded in the $CDCl_3$ as solvent. The chemical shift (δ) for ^{1}H NMR and ^{13}C NMR are given in ppm relative to residual signals of the solvents. Coupling constants are given in Hertz (Hz). The following abbreviations are used to indicate the multiplicity: s, singlet; d, doublet; t, triplet; q, quartet; p, pentet; sept, septet; m, multiplet. High-resolution mass spectra (HRMS) were obtained from the High-Resolution Mass Spectrometry using electrospray ionization time-of-flight (ESI-TOF) reflection experiments.

Optimization of the Reaction Condition

At the beginning, our investigated with the model reaction of iodobenzene **1a,** selenium powder **2** to study reaction conditions including the optimization of catalysts, bases and solvents. As shown in Table S1, at the outset, copper salts were used as catalyst (entries 1-6), no desired product was gained when the reaction conducted in the presence of CuO as the catalyst in DMSO (entry 1).

The experiment result shows proper solvent was critical for this reaction, when the reactions were conducted in apolar solvent such DMF product was detected moderate yield. $CuBr_2$ was proved to be the best efficient catalyst species in this reaction (entry 5). Gratifyingly, the yield of product **4a** was obtained in 75% when the catalyst changed to $Cu(OAc)_2$ (entry 6). By screening different bases for the reaction, Cs_2CO_3 was demonstrated to be more suitable base than others such as NaOH, Na_2CO_3, K_2CO_3 and Na_2SO_4 (entries 6-10). Reducing yield was obtained in the reaction operated in 100°C (72% yield, entry13) and 120 °C (77% yield, entry13). Finally determine the optimal reaction conditions were $Cu(OAc)_2$ as the catalyst, Cs_2CO_3 as the base, the ratio of **1a**:**2** was 1:1.5:1, under N_2, in 110 °C, preparation for 24 hours.

Table S1. Optimization of the reaction conditions.[a]

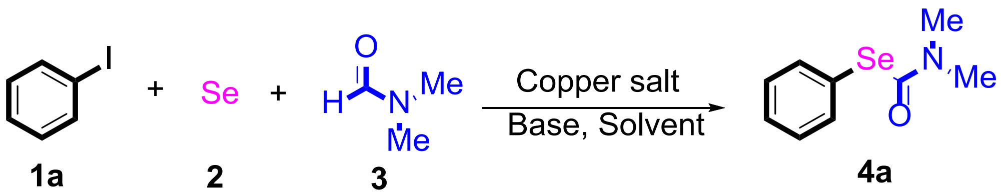

Entry	Copper salt	Base	Solvent	1a:2	Yield (%)[b]
1	CuO	Na_2CO_3	DMSO	1:1	0
2	CuSO4	Na_2CO_3	DMF	1:1	18
3	CuI	Na_2CO_3	DMF	1:1	29
4	$CuCl_2$	Na_2CO_3	DMF	1:1	34
5	$CuBr_2$	Na_2CO_3	DMF	1:1	60
6	$Cu(OAc)_2$	Na_2CO_3	DMF	1:1	75
7	$Cu(OAc)_2$	Cs_2CO_3	DMF	1:1	86
8	$Cu(OAc)_2$	NaOH	DMF	1:1	56

9	$Cu(OAc)_2$	Na_2SO_4	DMF	1:1	49
10	$Cu(OAc)_2$	NaOEt	DMF	1:1	65
11	Cu(OAc)2	K_2CO_3	DMF	1:1	55
12	Cu(OAc)2	K_2PO_3	DMF	1:1	57
13	$Cu(OAc)_2$	Cs_2CO_3	DMF	1:1	5
14	$Cu(OAc)_2$	Cs_2CO_3	DMF	1:1	48
15	$Cu(OAc)_2$	Cs_2CO_3	DMF	1:1.5	72[c]
16	$Cu(OAc)_2$	Cs_2CO_3	DMF	1:1.5	77[d]
17	$Cu(OAc)_2$	Cs_2CO_3	DMF	1:1.5	64[c]
18	$Cu(OAc)_2$	Cs_2CO_3	DMF	1:1.5	69[d]

[a] Unless otherwise noted, reactions conditions were iodobenzene **1a** (2.04 g, 10 mmol) and selenium powder **2** (1.19 g, 15 mmol), copper catalyst (10 mol%), base (2 equiv, under N_2 atmosphere), DMF (10 mL). The tube was evacuated and refilled with N_2 three times. The reaction is carried out under nitrogen protection. The reaction mixture was stirred at 110 ^{o}C for 12 h. After it was cooled, the reaction mixture was diluted with 20 mL of ethyl ether for 3 times. The filtrate was washed with water (3×15 mL). The organic phase was dried over Na_2SO_4, filtered, and concentrated under reduced pressure. and filtered through a pad of silica gel, followed by washing the pad of silica gel with the same solvent (20 mL). The residue was then purified by flash chromatography on silica gel to provide the corresponding product. The pure product Se-phenyl dimethylcarbamoselenoate (**4a**) was obtained 1.96 g, 86% yield.

[b] Isolated yield.

[c] 100 ^{o}C.

[d] 120 ^{o}C.

[e] $Cu(OAc)_2$ (15 mol%).

[f] $Cu(OAc)_2$ (5 mol%).

General Procedures for Preparation of 4 and 6

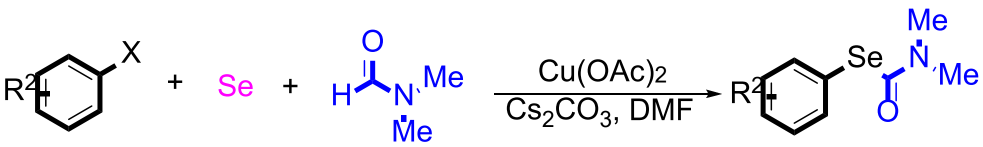

1, 5 **2** **3** **4, 6**

A mixture of iodobenzene **1a** (2.04 g, 10 mmol) and selenium powder **2** (0.79 g, 15 mmol), $Cu(OAc)_2$ (182 mg, 10 mol%), Cs_2CO_3 (6.52 g, 2 equiv), DMF (10 mL). The tube was evacuated and refilled with N_2 three times. The reaction is carried out under nitrogen protection. The reaction mixture was stirred at 110 ^{o}C for 12 h. After it was cooled, the reaction mixture was diluted with 20 mL of ethyl ether for 3 times. The filtrate was washed with water (3×15 mL). The organic phase was dried over Na_2SO_4, filtered, and concentrated under reduced pressure. and filtered through a pad of silica gel, followed by washing the pad of silica gel with the same solvent (20 mL). The residue was then purified by flash chromatography on silica gel to provide the corresponding product. The pure product Se-phenyl dimethylcarbamoselenoate **4a** was obtained 1.96 g, 86% yield.

Preliminary Mechanism Investigation

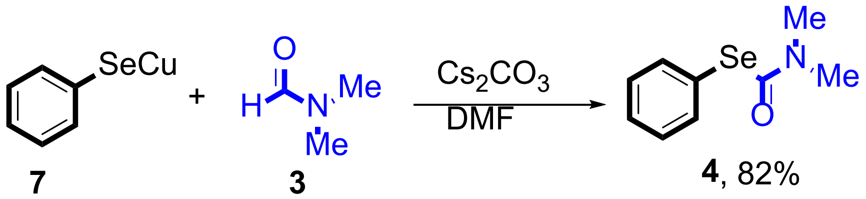

Scheme S2. Preliminary mechanism investigation.

To obtain the preliminary results of the reaction mechanism, some additional reactions were been done, Scheme 2. At first, a mixture of PhSeCu **7** (2.20 g, 10 mmol), $Cu(OAc)_2$ (182 mg, 10 mol%), Cs_2CO_3 (6.52 g, 2 equiv) and DMF (10 mL). The tube was evacuated and refilled with N_2 three times. The reaction is carried out under nitrogen protection. The reaction mixture was stirred at 110 ^{o}C for 12 h. After it was cooled, the reaction mixture was diluted with 20 mL of ethyl ether for 3 times. The filtrate was washed with water (3×15 mL). The organic phase was dried over Na_2SO_4, filtered, and concentrated under reduced pressure. and filtered through a pad of silica gel, followed by washing the pad of silica gel with the same solvent (20 mL). The residue was then purified by flash chromatography on silica gel to provide the corresponding product. The pure product Se-phenyl dimethylcarbamoselenoate **4a** was obtained 1.87 g, 86% yield.

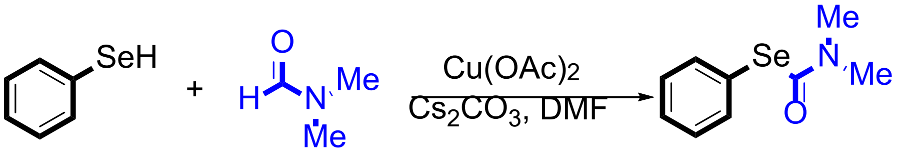

Scheme S3. Preliminary mechanism investigation.

A mixture of benzeneselenol **8** (1.57 g, 10 mmol) and $Cu(OAc)_2$ (182 mg, 10

mol%), Cs_2CO_3 (6.52 g, 2 equiv), DMF (10 mL). The tube was evacuated and refilled with N_2 three times. The reaction is carried out under nitrogen protection. The reaction mixture was stirred at 110 °C for 12 h. After it was cooled, the reaction mixture was diluted with 20 mL of ethyl ether for 3 times. The filtrate was washed with water (3×15 mL). The organic phase was dried over Na_2SO_4, filtered, and concentrated under reduced pressure. and filtered through a pad of silica gel, followed by washing the pad of silica gel with the same solvent (20 mL). The residue was then purified by flash chromatography on silica gel to provide the corresponding product. The pure product Se-phenyl dimethylcarbamoselenoate **4a** was obtained 1.82 g, 79% yield.

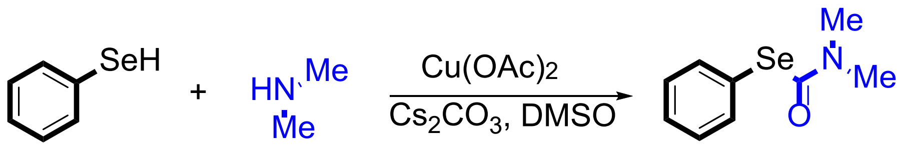

8 **9** **4**, nr

Scheme S4. Preliminary mechanism investigation.

A mixture of benzeneselenol **8** (1.57 g, 10 mmol) and $Cu(OAc)_2$ (182 mg, 10 mol%), Cs_2CO_3 (6.52 g, 2 equiv), dimethylamine (10 mL). The tube was evacuated and refilled with N_2 three times. The reaction is carried out under nitrogen protection. The reaction mixture was stirred at 110 °C for 12 h. After it was cooled, the reaction mixture was diluted with 20 mL of ethyl ether for 3 times. The filtrate was washed with water (3×15 mL). The organic phase was dried over Na_2SO_4, filtered, and concentrated under reduced pressure. and filtered through a pad of silica gel, followed by washing the pad of silica gel with the same solvent (20 mL). The residue was then purified by flash

chromatography on silica gel to provide nothing of the corresponding product.

Analytical Data

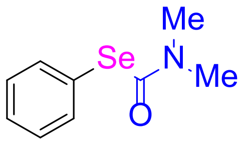

4a

Se-phenyl dimethylcarbamoselenoate (4a) Pale Yellow oil liquid, 1.96 g, 86% yield;

^{1}H NMR (400 MHz, $CDCl_3$) δ (ppm): 7.41-7.37 (m, 2H), 7.27-7.23 (m, 1H), 7.08-7.06 (m, 2H), 3.44 (s, 3H), 3.33(s, 3H); ^{13}C NMR (100 MHz, $CDCl_3$) δ (ppm): 187.9, 154.1, 129.2, 125.9, 122.8, 43.3, 38.8; HRMS ESI: Calcd. m/z for $C_9H_{11}NNaOSe$ $[M+Na]^+$ 251.9904, found 251.9900.

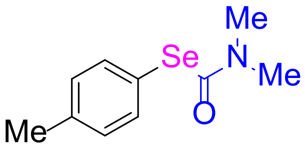

4b

Se-(p-tolyl) dimethylcarbamoselenoate (4b) Pale Yellow oil liquid, 2.03 g, 84% yield;

^{1}H NMR (400 MHz, $CDCl_3$) δ (ppm): 7.30 (m, 2H), 7.12 (m, 2H), 2.97 (m, 6H), 2.28 (s, 3H); ^{13}C NMR (100 MHz, $CDCl_3$) δ (ppm): 167.3, 139.4, 135.7, 129.7, 125.2, 36.9, 21.3; HRMS ESI: Calcd. m/z for $C_{10}H_{13}NNaOSe$ $[M+Na]^+$ 266.0060, found 266.0057.

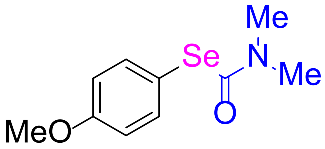

4c

Se-(4-methoxyphenyl) dimethylcarbamoselenoate (4c) Pale Yellow oil liquid,

2.09 g, 81% yield;

^{1}H NMR (400 MHz, $CDCl_3$) δ (ppm): 7.43 (m, 2H), 6.94 (m, 2H), 3.83 (s, 3H), 3.07 (s, 6H); ^{13}C NMR (100 MHz, $CDCl_3$) δ (ppm): 167.3, 160.5, 135.7,137.3, 119.4, 114.6, 55.3, 36.8; HRMS ESI: Calcd. m/z for $C_{10}H_{13}NNaO_2Se$ $[M+Na]^+$ 282.0009, found 282.0006.

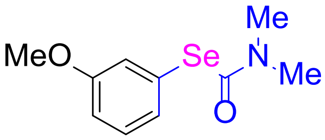

4d

Se-(3-methoxyphenyl) dimethylcarbamoselenoate (4d) Pale Yellow oil liquid, 2.14 g, 83% yield;

^{1}H NMR (400 MHz, $CDCl_3$) δ (ppm): 7.30 (m, 1H), 6.81-6.78 (m, 1H), 6.68 (m, 1H), 6.66 (m, 1H), 3.80 (s, 3H), 3.45 (s, 3H), 3.33 (s, 3H); ^{13}C NMR (100 MHz, $CDCl_3$) δ (ppm): 187.7, 160.3, 155.0, 129.5, 115.0, 111.8, 108.9, 55.4, 43.3, 38.7; HRMS ESI: Calcd. m/z for $C_{10}H_{13}NNaO_2Se$ $[M+Na]^+$ 282.0009, found 282.0006.

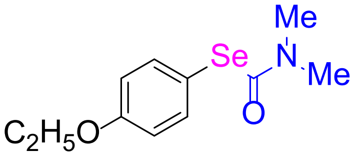

4e

Se-(4-ethoxyphenyl) dimethylcarbamoselenoate (4e) Pale Yellow oil liquid, 2.18 g, 80% yield;

^{1}H NMR (400 MHz, $CDCl_3$) δ (ppm): 7.42 (m, 2H), 6.93 (m, 2H), 4.08 (m, 2H), 3.45 (m, 4H), 1.45 (m, 3H), 1.28 (m, 3H), 1.19 (s, 3H); ^{13}C NMR (100 MHz, $CDCl_3$) δ (ppm): 166.5, 159.9, 137.4, 119.3, 115.1, 63.6, 42.3, 14.8, 13.8, 13.2; HRMS ESI: Calcd. m/z for $C_{11}H_{15}NNaO_2Se$ $[M+Na]^+$ 296.0166, found 296.0163.

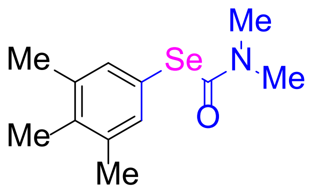

4f

Se-(3,4,5-trimethylphenyl) dimethylcarbamoselenoate (4f) Pale Yellow oil liquid, 2.13 g, 79% yield;

^{1}H NMR (400 MHz, $CDCl_3$) δ (ppm): 6.99 (m, 2H), 3.17 (m, 3H), 3.05 (m, 3H), 2.40 (s, 6H), 2.30 (m, 6H); ^{13}C NMR (100 MHz, $CDCl_3$) δ (ppm): 166.3, 143.4, 139.5, 129.1, 124.7, 37.0, 21.9, 21.2; HRMS ESI: Calcd. m/z for $C_{12}H_{17}NNaOSe$ $[M+Na]^+$ 294.0373, found 294.0370.

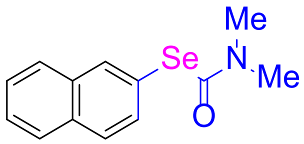

4g

Se-(naphthalen-2-yl) dimethylcarbamoselenoate (4g) Pale Yellow oil liquid, 2.56 g, 92% yield;

^{1}H NMR (400 MHz, $CDCl_3$) δ (ppm): 8.02 (s, 1H), 7.85-7.80 (m, 3H), 7.50-7.45 (m, 3H), 3.13 (s, 3H), 3.05 (s, 3H); ^{13}C NMR (100 MHz, $CDCl_3$) δ (ppm): 167.0, 135.3, 133.5, 133.3, 132.3, 128.5, 128.0, 127.7, 126.9, 126.3, 126.1, 37.0; HRMS ESI: Calcd. m/z for $C_{13}H_{13}NNaOSe$ $[M+Na]^+$ 302.0060, found 302.0057.

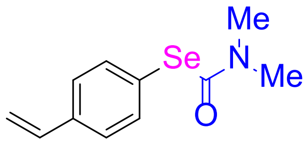

4h

Se-(4-vinylphenyl) dimethylcarbamoselenoate (4h) Pale Yellow oil liquid, 2.29 g, 90% yield;

^{1}H NMR (400 MHz, $CDCl_3$) δ (ppm): 7.39 (m, 1H), 6.84 (m, 2H), 6.03 (s, 1H), 5.15-5.08 (m, 2H), 3.88 (s, 3H), 3.42 (m, 2H), 3.13 (s, 3H), 3.02 (s, 3H); ^{13}C NMR (100 MHz, $CDCl_3$) δ (ppm): 166.6, 160.1, 144.2, 138.1, 136.7, 121.3, 116.4, 114.1, 111.9, 56.1, 40.4, 37.0; HRMS ESI: Calcd. m/z for $C_{11}H_{13}NNaOSe$ $[M+Na]^+$ 278.0060, found 278.0057.

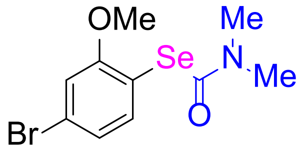

4i

Se-(4-bromo-2-methoxyphenyl) dimethylcarbamoselenoate (4i) Pale Yellow oil liquid, 3.00 g, 88% yield;

^{1}H NMR (400 MHz, $CDCl_3$) δ (ppm): 7.32-7.29 (m, 1H), 7.12-7.07 (m, 2H), 3.86 (s, 3H), 3.10 (s, 3H), 3.01 (s, 3H); ^{13}C NMR (100 MHz, $CDCl_3$) δ (ppm): 165.6, 160.5, 138.9, 125.1, 124.1, 116.2, 115.2, 56.4, 37.0; HRMS ESI: Calcd. m/z for $C_{10}H_{12Br}NNaOSe$ $[M+Na]^+$ 359.9114, found 359.9111.

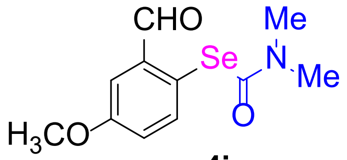

4j

Se-(2-formyl-4-methoxyphenyl) dimethylcarbamoselenoate (4j) Pale Yellow oil liquid, 2.55 g, 89% yield;

^{1}H NMR (400 MHz, $CDCl_3$) δ (ppm): 7.53 (s, 1H), 7.46 (m, 1H), 7.14 (d, J = 8.5 Hz, 1H), 3.87 (s, 3H), 3.15 (s, 3H), 3.02 (s, 3H); ^{13}C NMR (100 MHz, $CDCl_3$) δ (ppm): 191.2, 165.9, 161.1, 139.0, 138.9, 123.5, 121.2, 112.1, 55.7, 37.1; HRMS ESI: Calcd. m/z for $C_{11}H_{13}NNaO_3Se$ $[M+Na]^+$ 309.9958, found 309.9955.

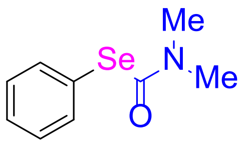

6a

Se-phenyl dimethylcarbamoselenoate (6a) Pale Yellow oil liquid, 1.85 g, 81% yield;

^{1}H NMR (400 MHz, $CDCl_3$) δ (ppm): 7.41 (m, 2H), 7.27 (m, 1H), 7.08 (m, 2H), 3.44 (s, 3H), 3.33 (s, 3H); ^{13}C NMR (100 MHz, $CDCl_3$) δ (ppm): 187.9, 154.1, 129.2, 125.9, 122.8, 43.3, 38.8; HRMS ESI: Calcd. m/z for $C_9H_{11}NNaOSe$ $[M+Na]^+$ 251.9904, found 251.9900.

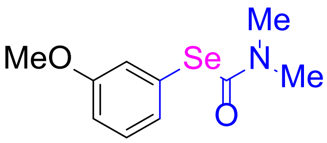

6b

Se-(3-methoxyphenyl) dimethylcarbamoselenoate (6b) Pale Yellow oil liquid, 2.01 g, 78% yield;

^{1}H NMR (400 MHz, $CDCl_3$) δ (ppm): 7.42 (m, 2H), 6.93 (m, 2H), 4.08 (m, 2H), 3.47 (m, 4H), 1.45 (m, 3H), 1.28 (m, 6H); ^{13}C NMR (100 MHz, $CDCl_3$) δ (ppm): 166.5, 159.8, 137.3, 119.2, 115.1, 63.5, 42.3, 14.8, 13.7, 13.2; HRMS ESI: Calcd. m/z for $C_{10}H_{13}NNaO_2Se$ $[M+Na]^+$ 282.0009, found 282.0006.

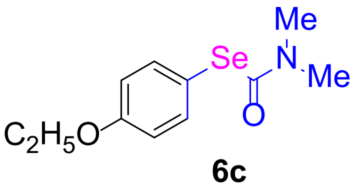

6c

Se-(4-ethoxyphenyl) dimethylcarbamoselenoate (6c) Pale Yellow oil liquid, 2.07 g, 76% yield;

^{1}H NMR (400 MHz, $CDCl_3$) δ (ppm): 7.42 (d, J = 8.8 Hz, 2H), 6.92 (d, J = 8.8 Hz,

2H), 4.06 (q, J = 7.0 Hz, 2H), 3.45 (m, 4H), 1.43 (m, 3H), 1.28 (s, 3H), 1.19 (s, 3H); ^{13}C NMR (100 MHz, $CDCl_3$) δ (ppm): 166.5, 159.9, 137.4, 119.3, 115.1, 63.6, 42.3, 14.8, 13.8, 13.2; HRMS ESI: Calcd. m/z for $C_{11}H_{15}NNaO_2Se$ $[M+Na]^+$ 296.0166, found 296.0163.

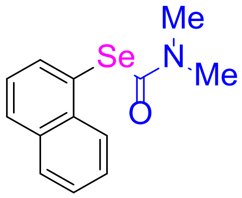

6d

Se-(naphthalen-1-yl) dimethylcarbamoselenoate(6d) Pale Yellow oil liquid, 2.36 g, 85% yield;

^1H NMR (400 MHz, $CDCl_3$) δ (ppm): 8.37 m, 1H,), 7.95 (m, 1H), 7.91 (m, 1H), 7.82 (m, 1H), 7.61 (m, 1H), 7.57 (m, 2H), 3.25 (m, 3H), 3.05 (m, 3H); ^{13}C NMR (100 MHz, $CDCl_3$) δ (ppm): 166.5, 136.1, 135.2, 134.2, 130.8, 128.6, 127.0, 126.22, 126.18, 125.8, 125.6, 37.0; HRMS ESI: Calcd. m/z for $C_{13}H_{13}NNaOSe$ $[M+Na]^+$ 302.0060, found 302.0057.

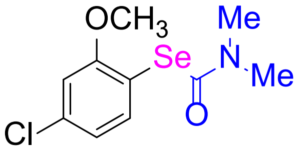

6e

Se-(4-chloro-2-methoxyphenyl) dimethylcarbamoselenoate (6e) Pale Yellow oil liquid, 2.72 g, 86% yield;

^1H NMR (400 MHz, $CDCl_3$) δ (ppm): 7.32-7.30 (m, 1H), 7.12-7.08 (m, 2H), 3.86 (s, 3H), 3.11 (s, 3H), 3.02 (s, 3H); ^{13}C NMR (100 MHz, $CDCl_3$) δ (ppm): 165.6, 160.6, 138.9, 125.2, 124.1, 116.2, 115.2, 56.4, 37.0; HRMS ESI: Calcd. m/z for $C_{10}H_{12}ClNNaO_2Se$ $[M+Na]^+$ 315.9619, found 315.9616.

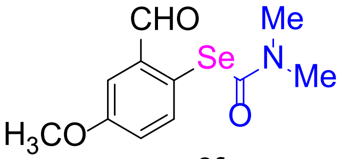

6f

Se-(2-formyl-4-methoxyphenyl) dimethylcarbamoselenoate (6f) Pale Yellow oil liquid, 2.37g, 83% yield;

^{1}H NMR (400 MHz, $CDCl_3$) δ (ppm): 7.53 (m, 1H), 7.46 (m, 1H), 7.71 (m, 1H), 3.87 (s, 3H), 3.16 (s, 3H), 3.01 (s, 3H); ^{13}C NMR (100 MHz, $CDCl_3$) δ (ppm): 191.2, 165.9, 161.1, 139.0, 138.9, 123.5, 121.2, 112.1, 55.7, 37.1; HRMS ESI: Calcd. m/z for $C_{11}H_{13}NNaO_3Se$ $[M+Na]^+$ 309.9958, found 309.9955.

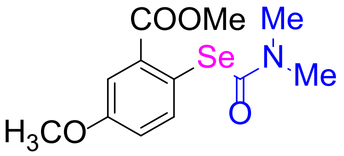

6g

methyl 2-((dimethylcarbamoyl)selanyl)-5-methoxybenzoate (6h) Pale Yellow oil liquid, 2.28 g, 72% yield;

^{1}H NMR (400 MHz, $CDCl_3$) δ (ppm): 7.50 (m, 1H), 7.40 (m, 1H), 7.03 (m, 1H), 3.87 (s, 3H), 3.84 (s, 3H), 3.05 (s, 6H); ^{13}C NMR (100 MHz, $CDCl_3$) δ (ppm): 166.9, 160.1, 139.2, 136.6, 120.1, 117.4, 115.9, 55.5, 52.2, 36.9; HRMS ESI: Calcd. m/z for $C_{12}H_{15}NNaO_4Se$ $[M+Na]^+$ 340.0064, found 340.0061.

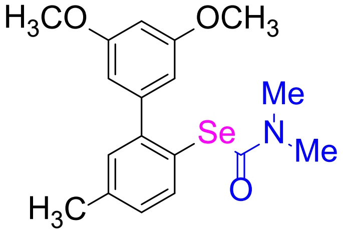

6h

Se-(3',5'-dimethoxy-5-methyl-[1,1'-biphenyl]-2-yl) dimethylcarbamoselenoate (2h) Pale Yellow oil liquid, 2.65 g, 70% yield;

^{1}H NMR (600 MHz, $CDCl_3$) δ (ppm): 7.50 (d, *J* = 7.6 Hz, 1H), 7.21 (d, *J* = 2.0 Hz, 1H), 7.20 (m, 1H), 6.52 (d, *J* = 2.3 Hz, 2H), 6.46 (t, *J* = 2.3 Hz, 1H), 3.78(s, 6H), 2.97 (s, 6H), 2.40 (s, 3H); ^{13}C NMR (150 MHz, $CDCl_3$) δ (ppm): 167.8, 160.1, 146.9, 143.5, 139.9, 138.1, 131.3, 129.0, 123.8, 107.6, 100.0, 55.5, 37.1, 21.4; HRMS ESI: Calcd. m/z for $C_{18}H_{21}NNaO_3Se$ $[M+Na]^+$ 402.0584, found 402.0581.

Spectrum

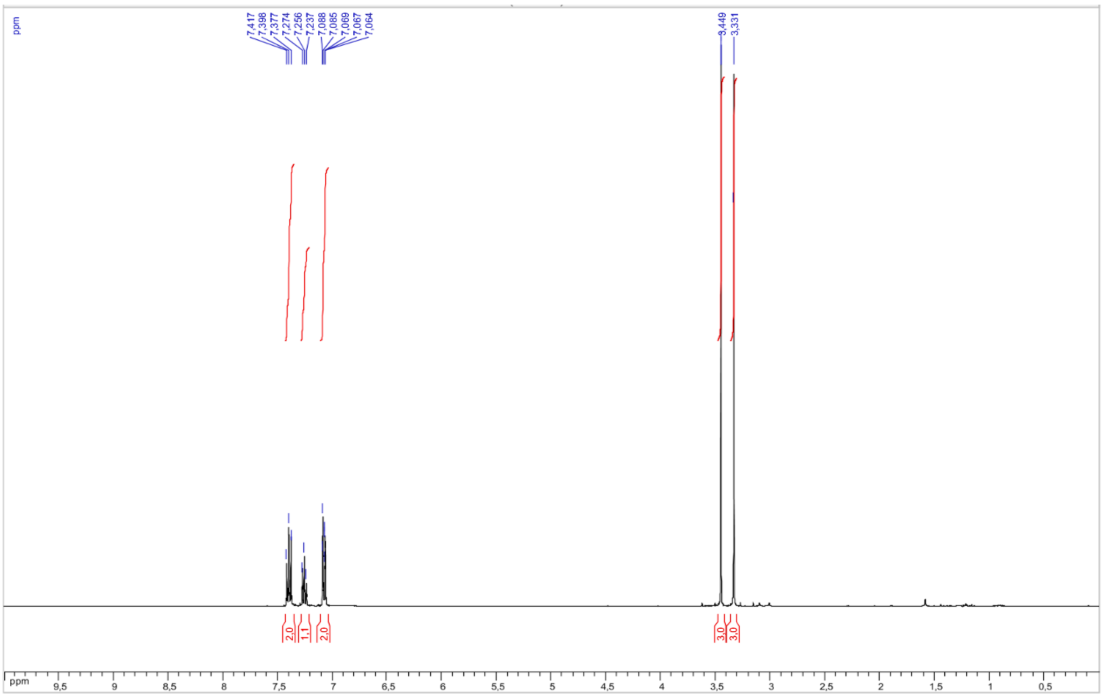

Figure S1. 4a ^{1}H NMR.

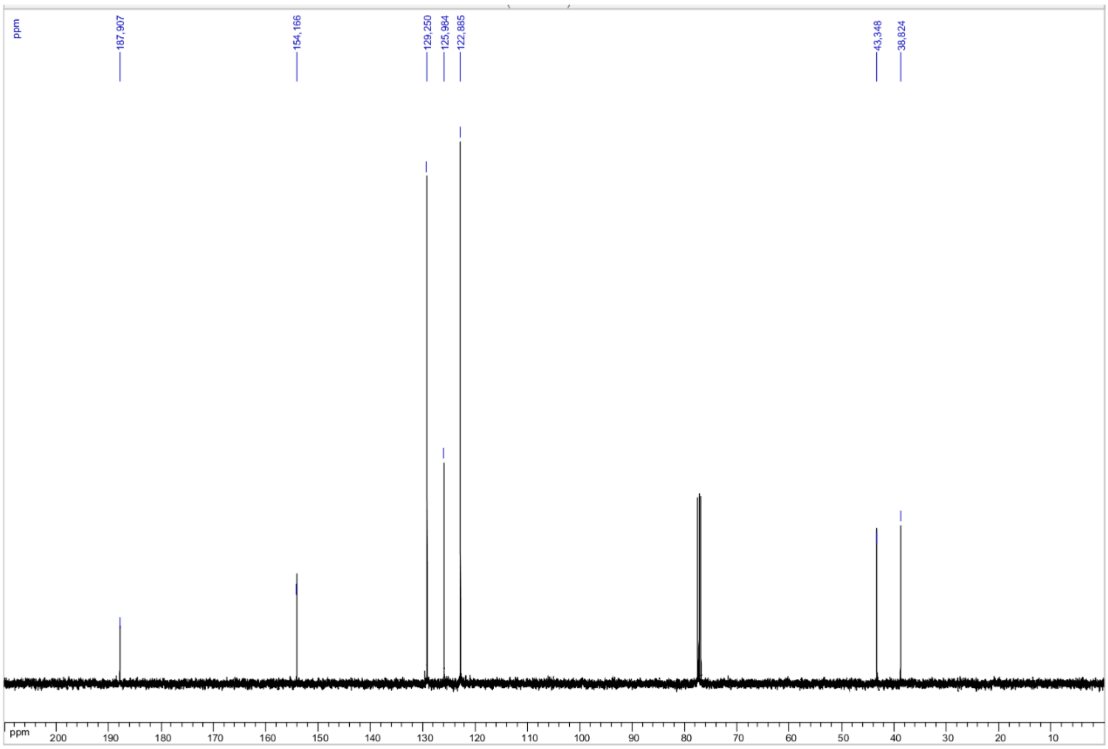

Figure S2. **4a** ^{13}C NMR

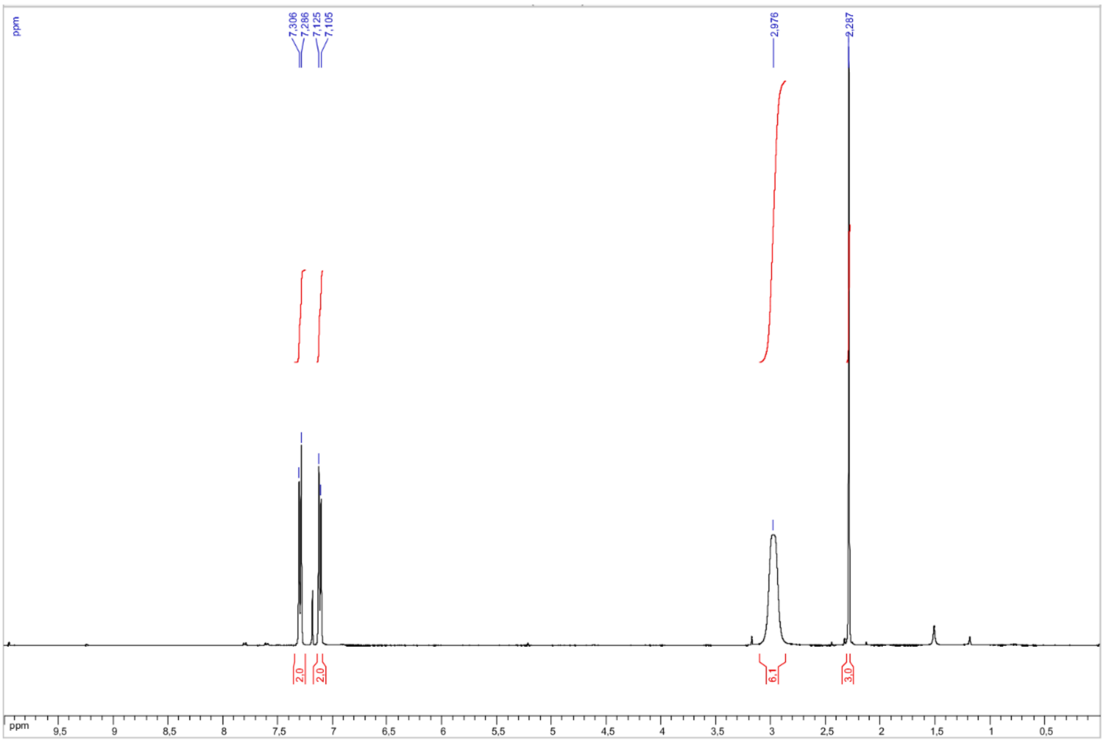

Figure S3. 4b ^{1}H NMR.

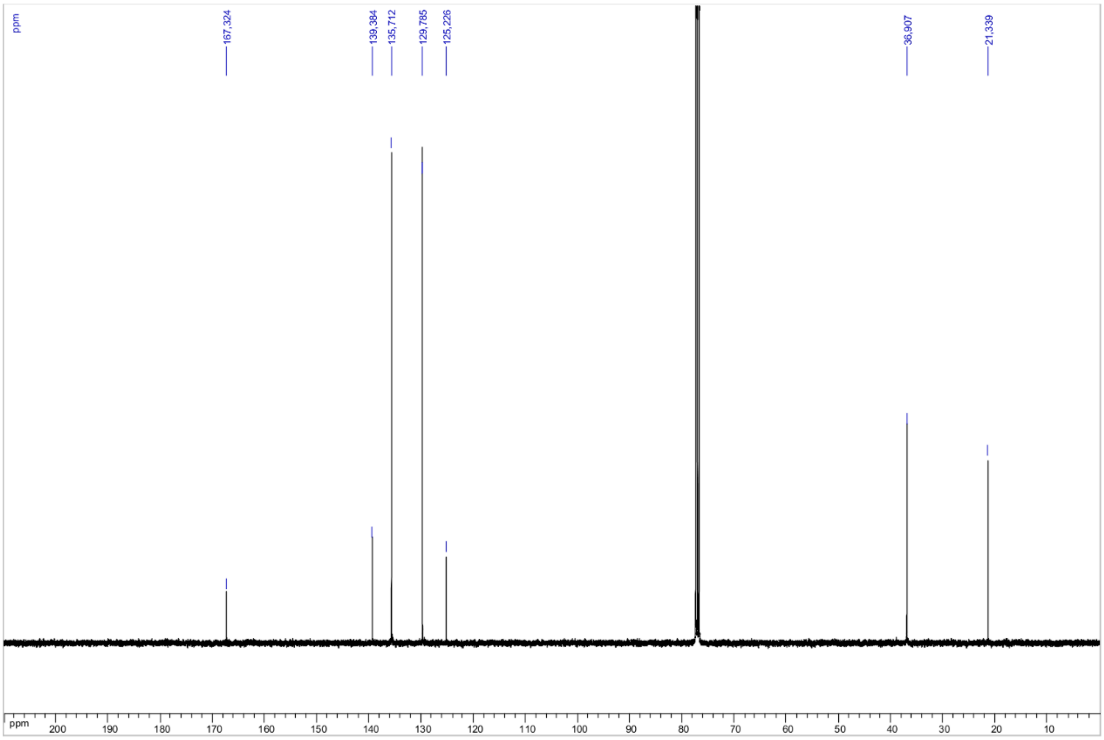

Figure S4. 4b ^{13}C NMR.

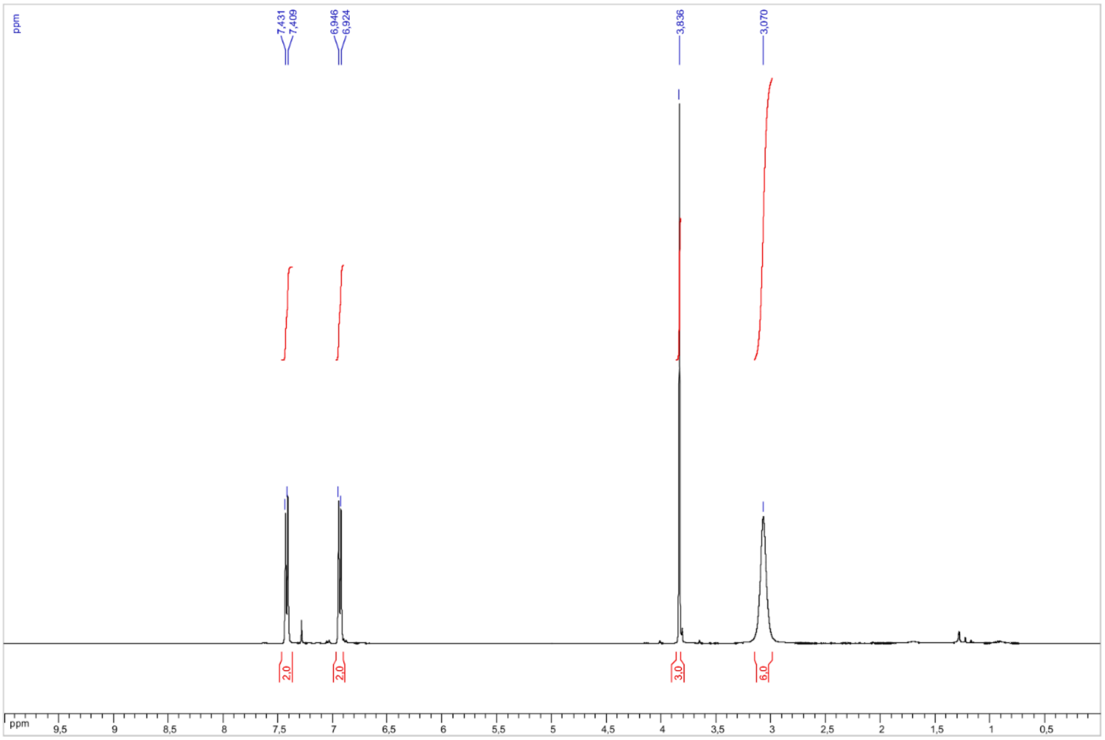

Figure S5. 4c ^{1}H NMR.

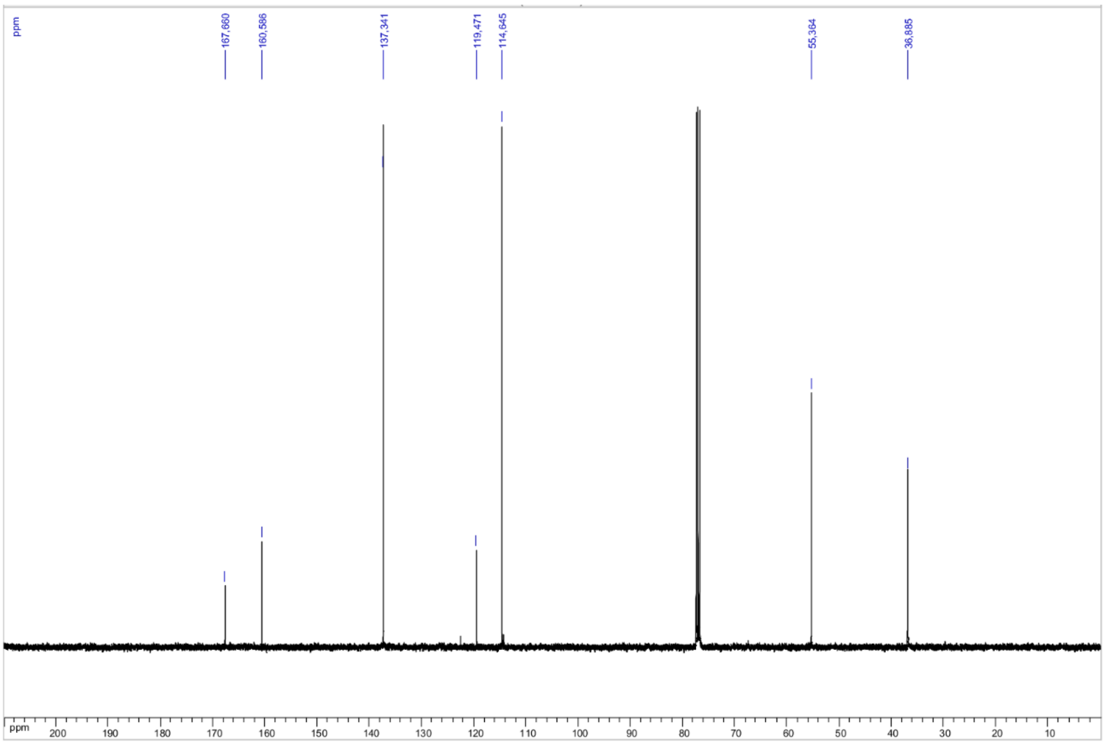

Figure S6. 4c ^{13}C NMR.

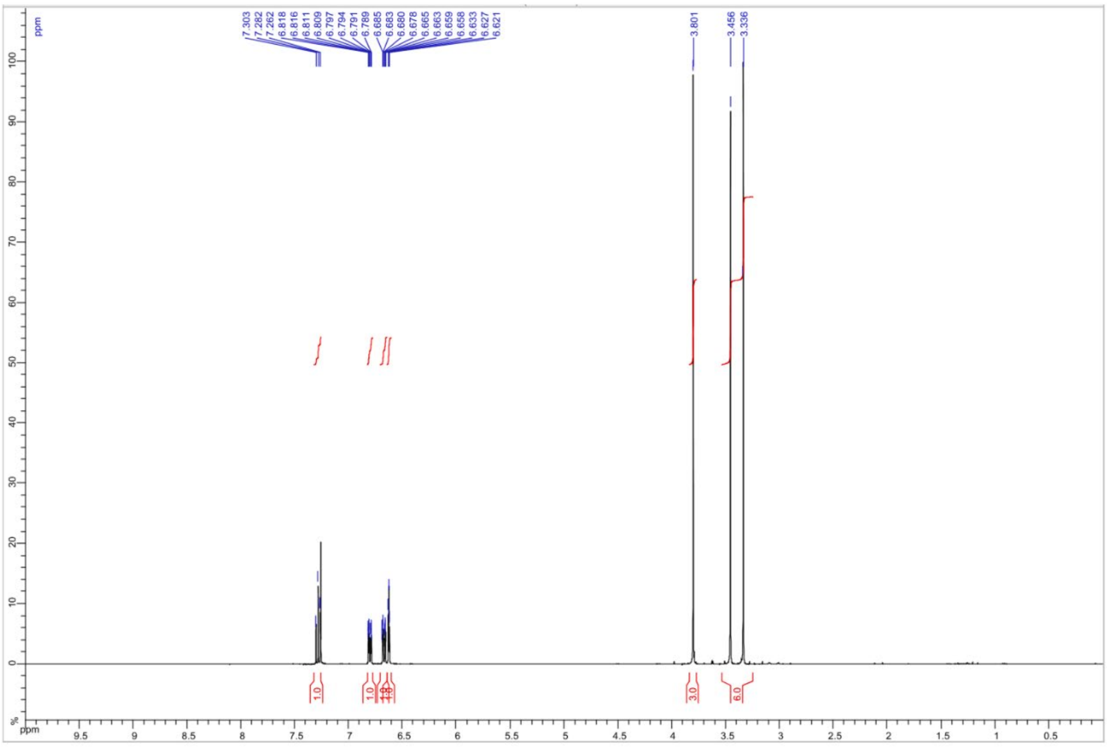

Figure S7. 4d ^{1}H NMR.

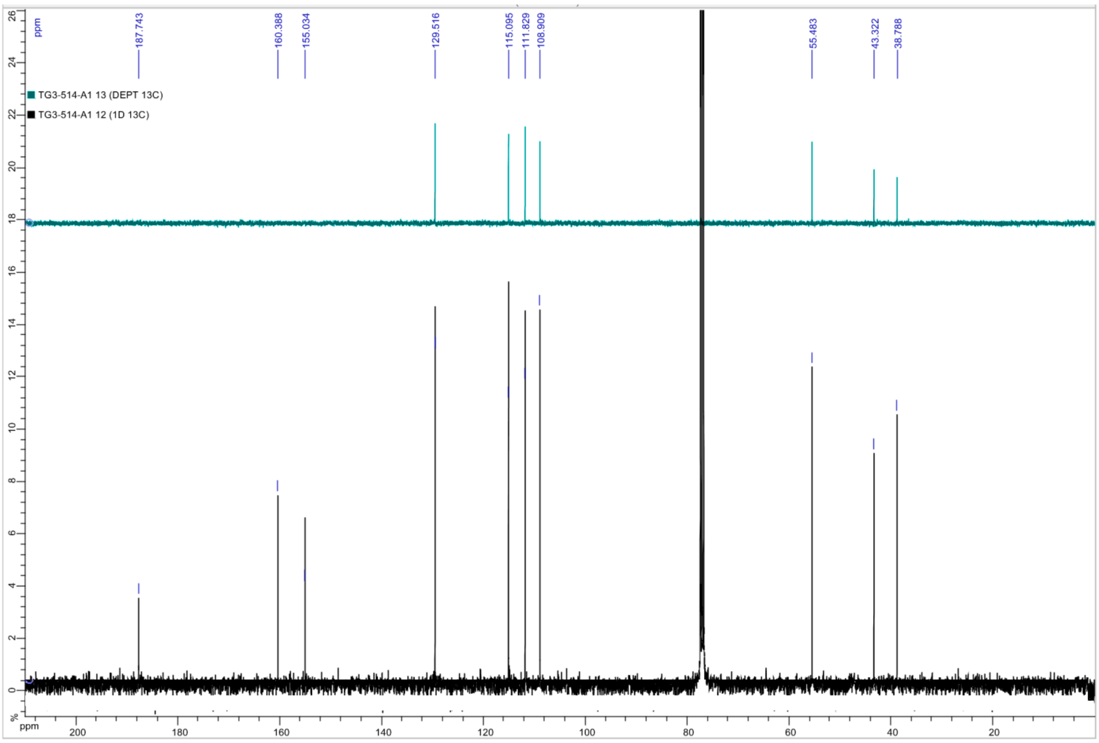

Figure S8. 4d ^{13}C NMR.

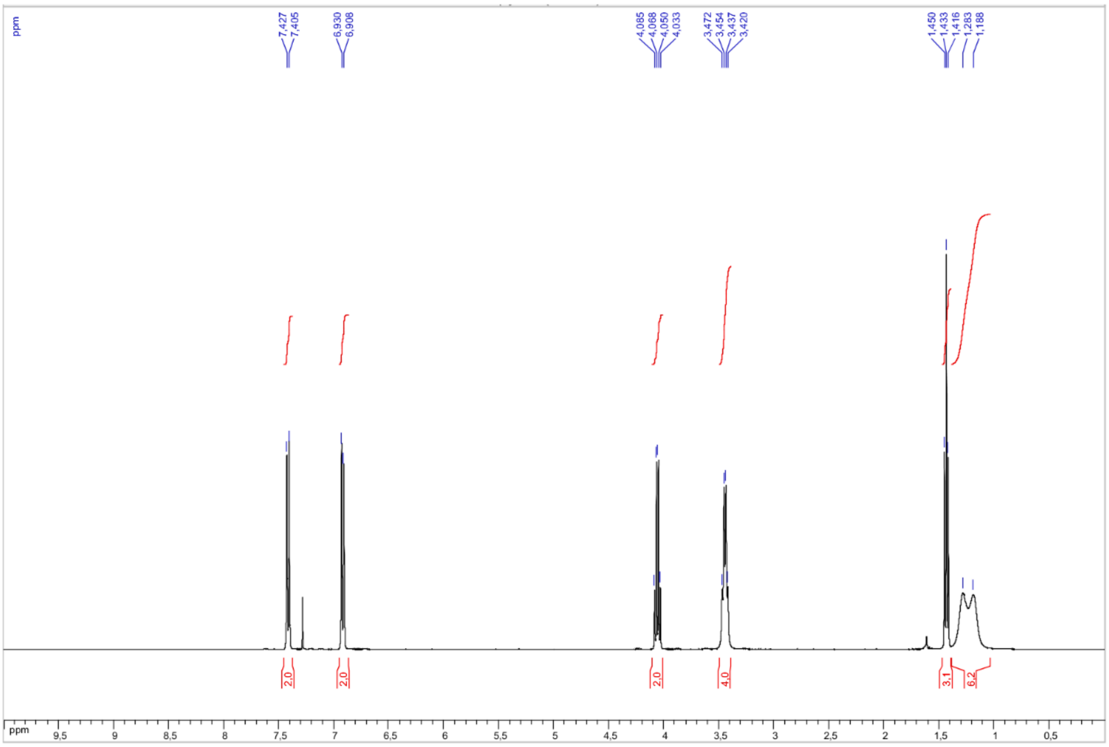

Figure S9. 4e ^{1}H NMR.

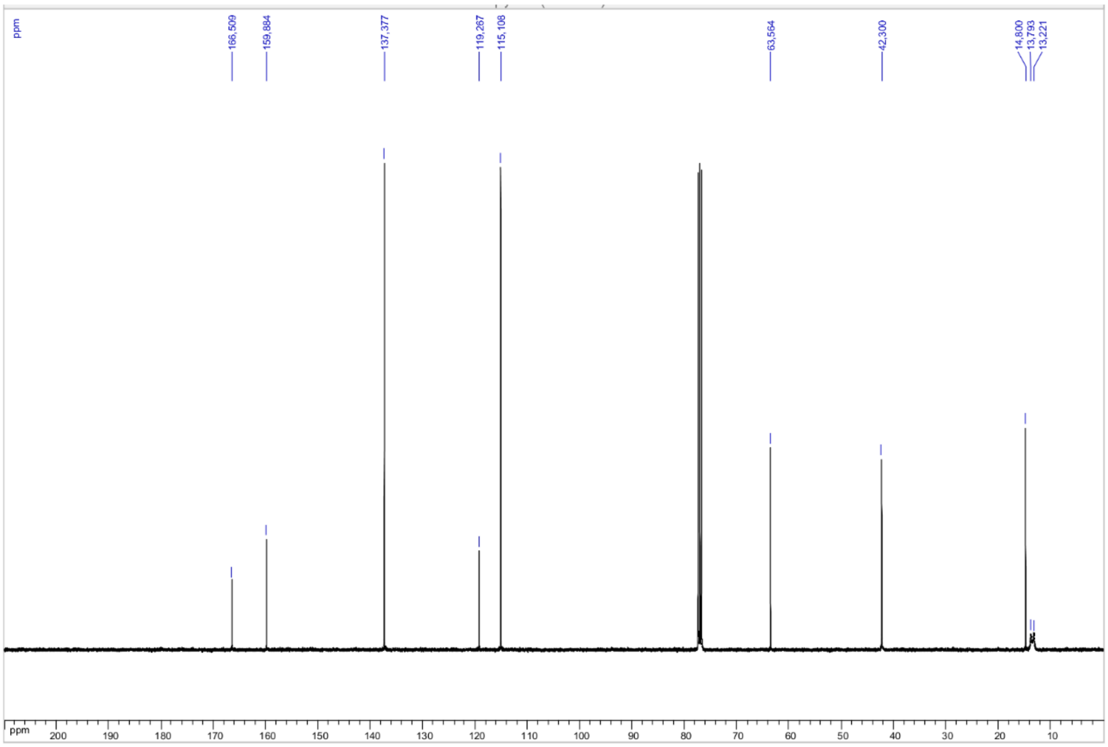

Figure S10. 4e ^{13}C NMR.

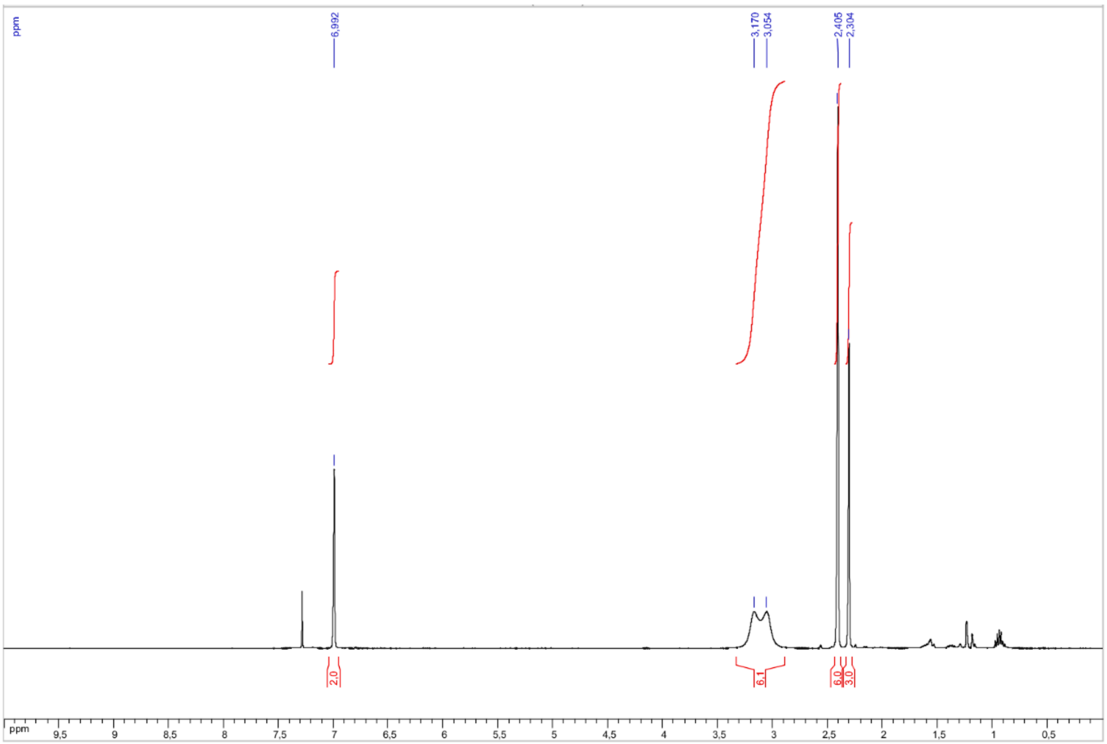

Figure S11. 4f ^{1}H NMR

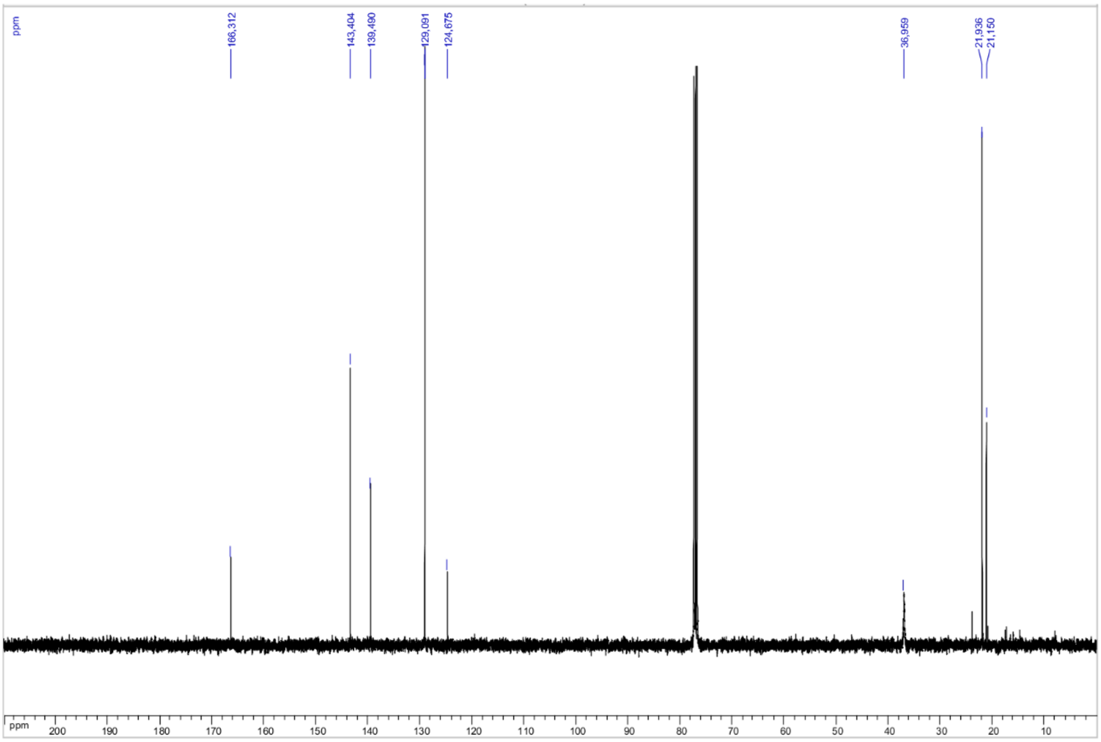

Figure S12. 4f ^{13}C NMR.

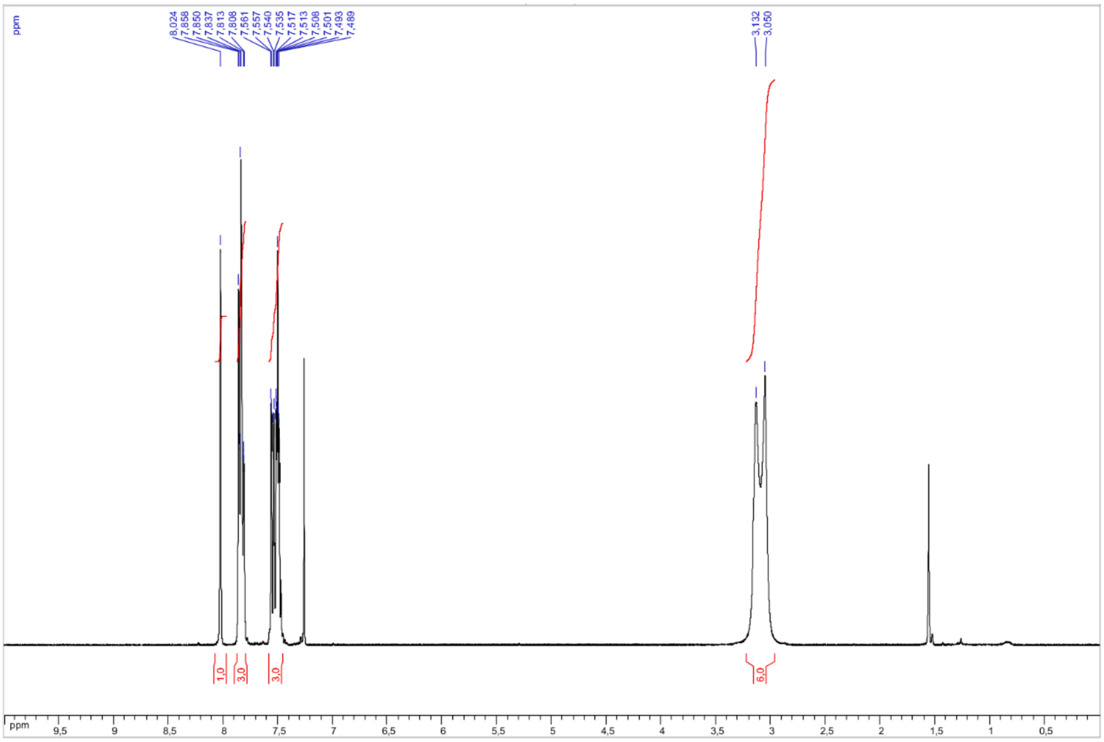

Figure S13. 4g ^{1}H NMR.

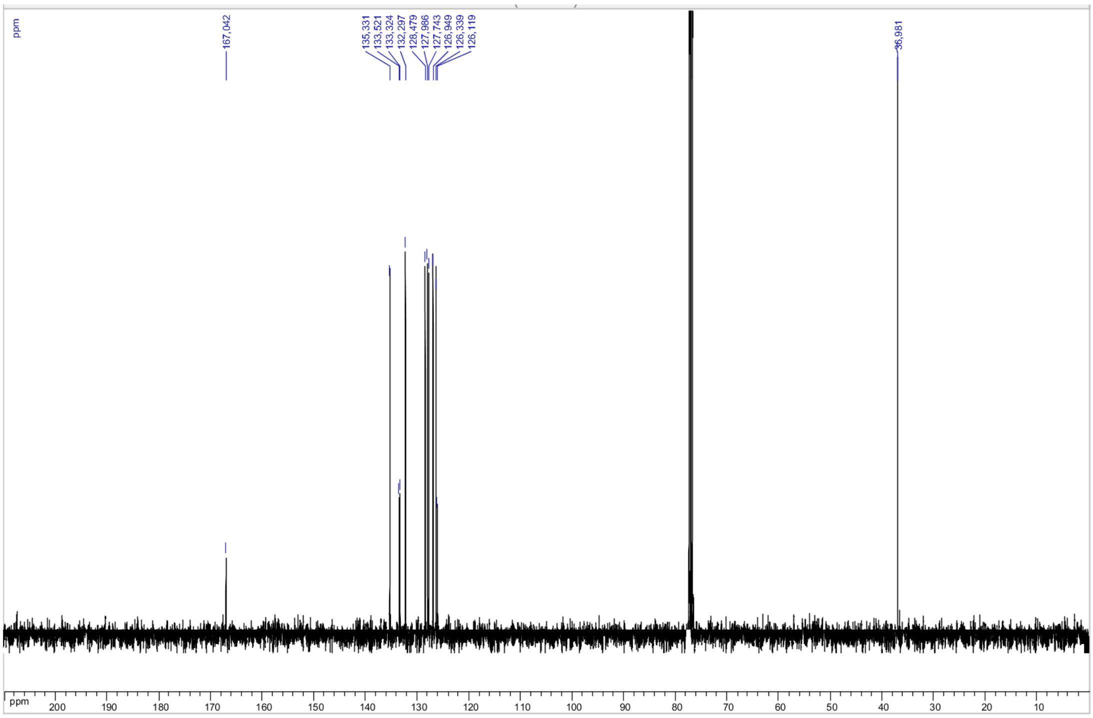

Figure S14. 4g ^{13}C NMR.

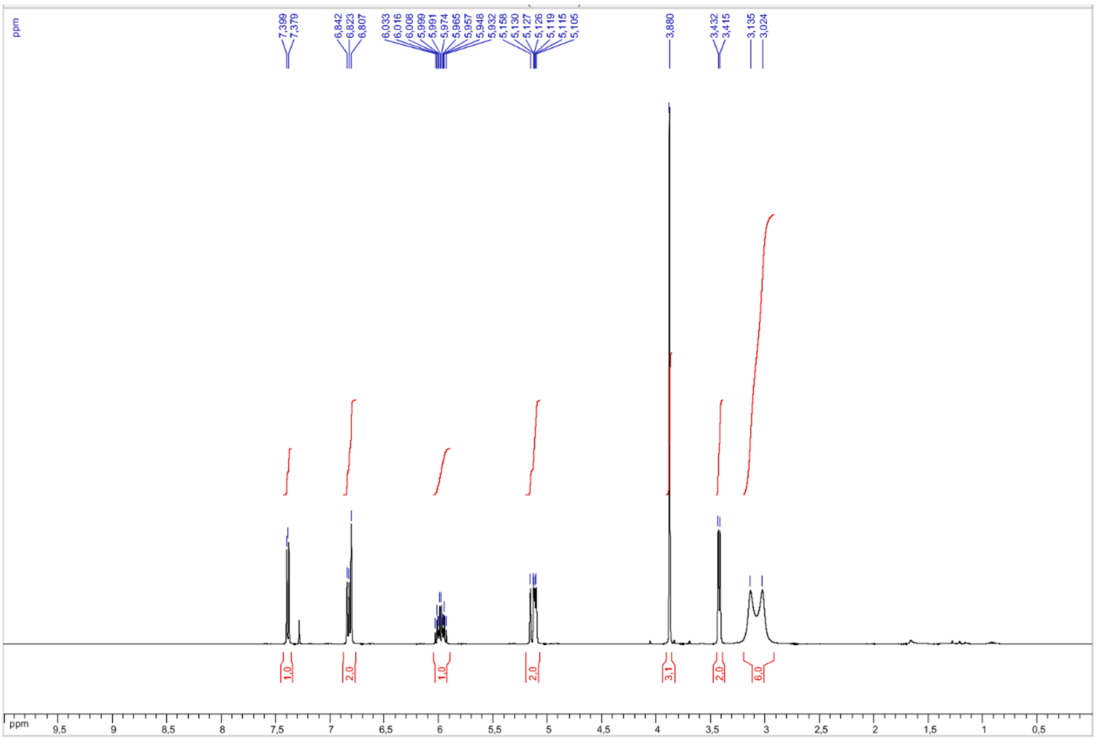

Figure S15. 4h ^{1}H NMR.

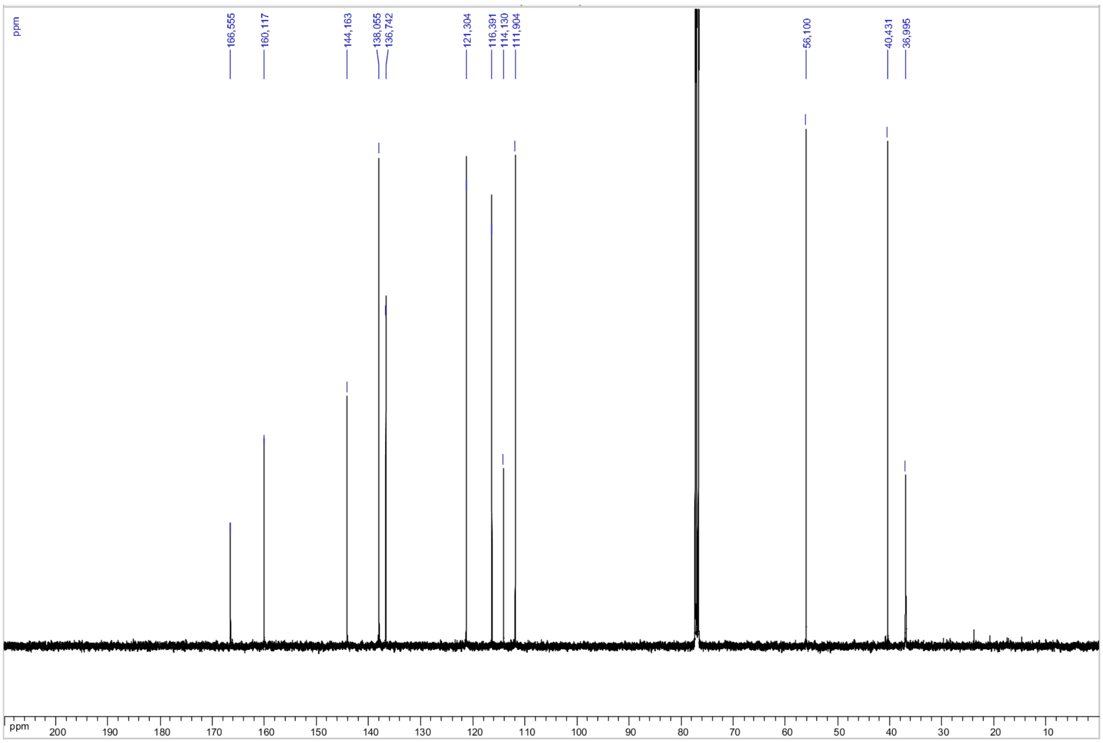

Figure S16. 4h ^{13}C NMR.

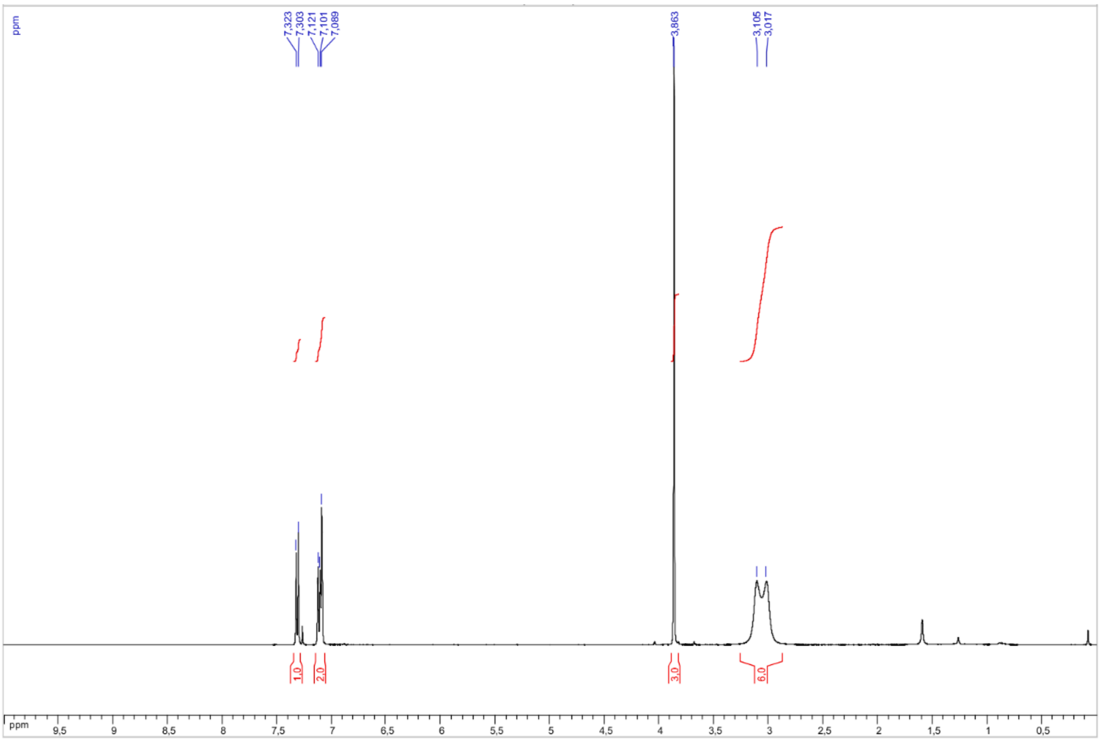

Figure S17. 4i ^{1}H NMR.

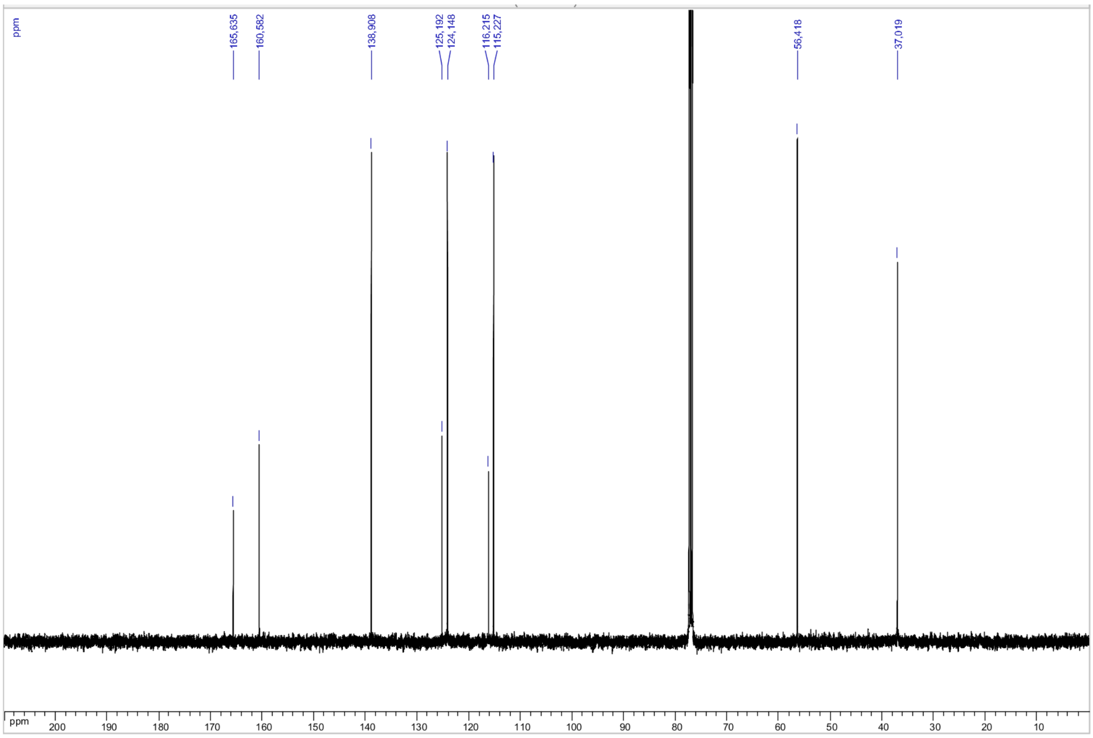

Figure S18. 4i ^{13}C NMR.

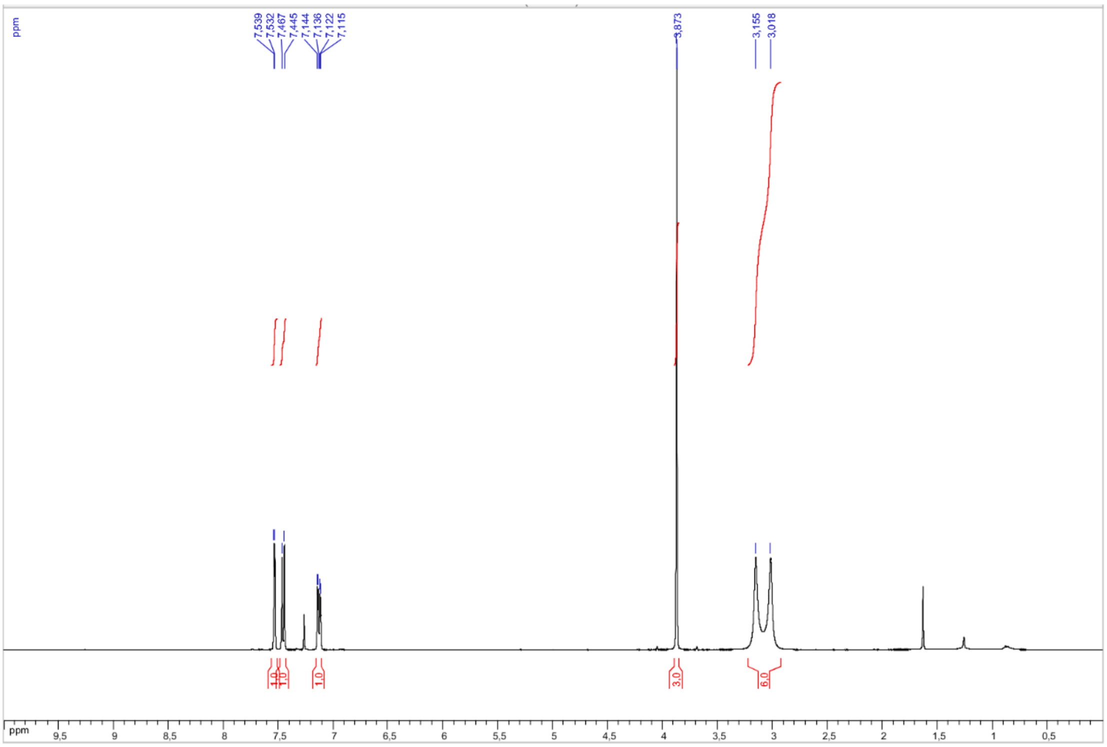

Figure S19. 4j ^{1}H NMR.

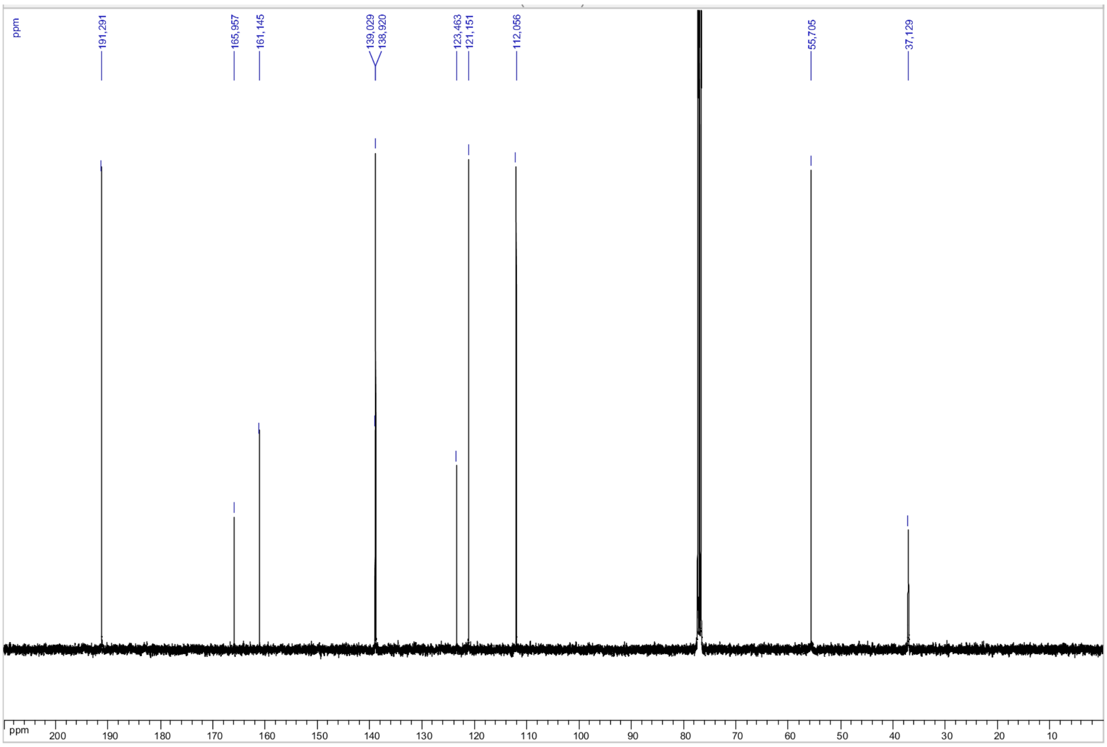

Figure S20. 4j ^{13}C NMR.

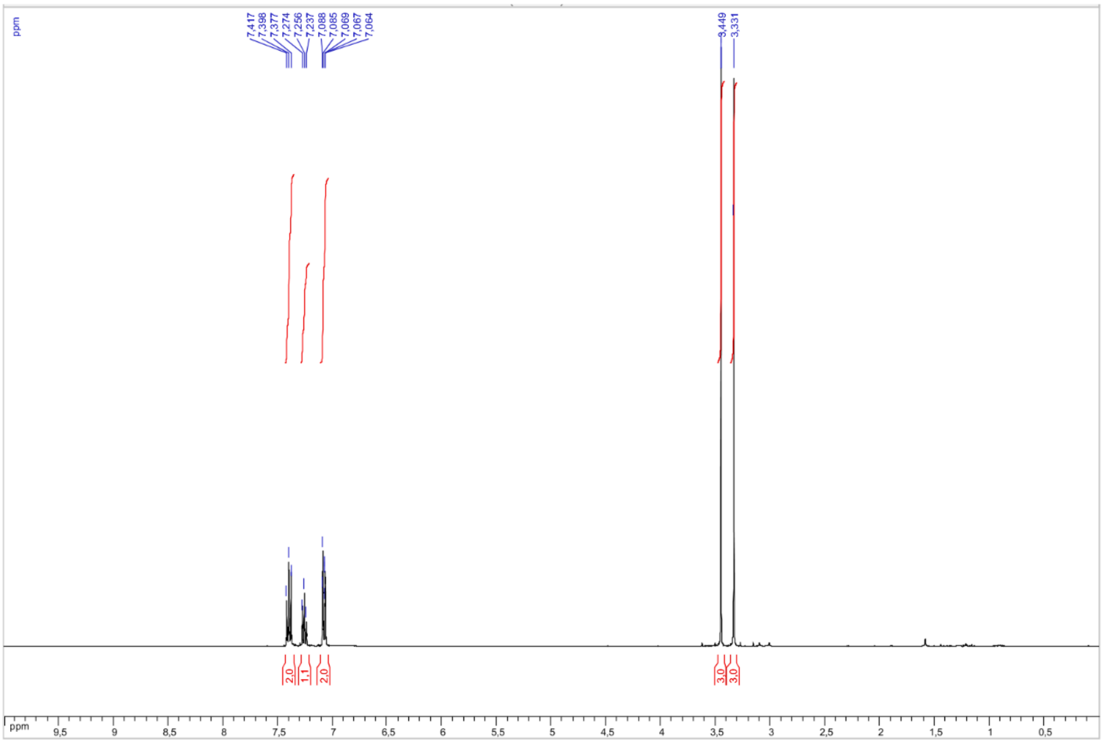

Figure S21. 6a ^{1}H NMR.

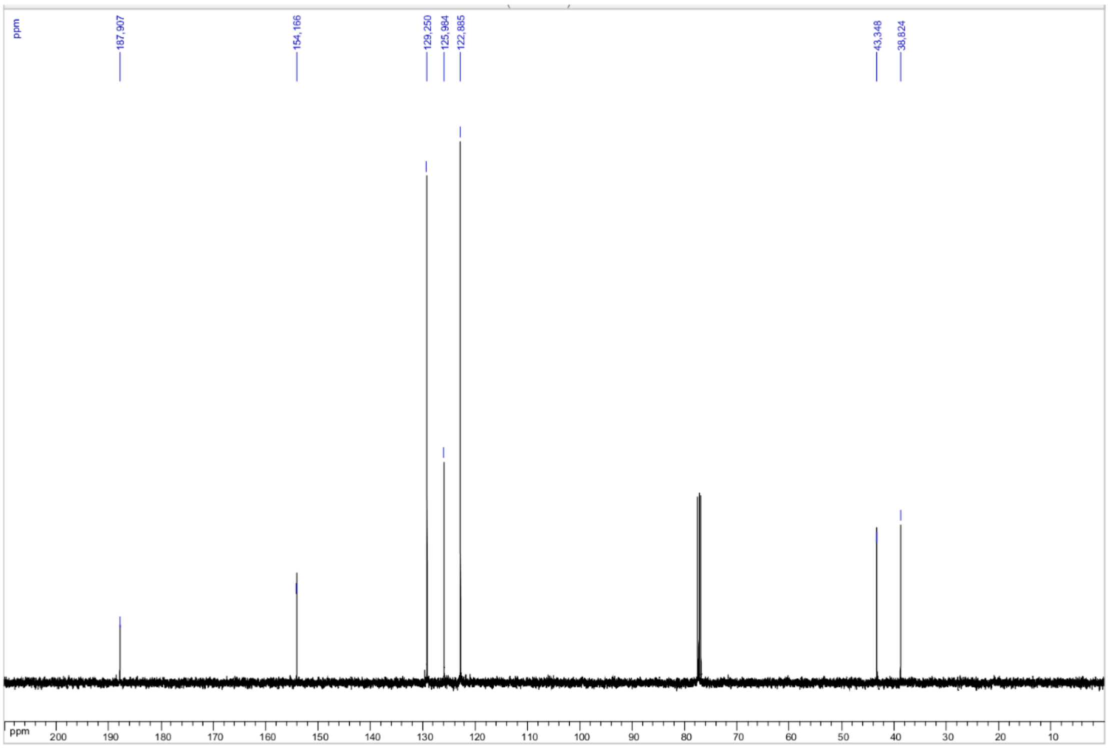

Figure S22. 6a ^{13}C NMR.

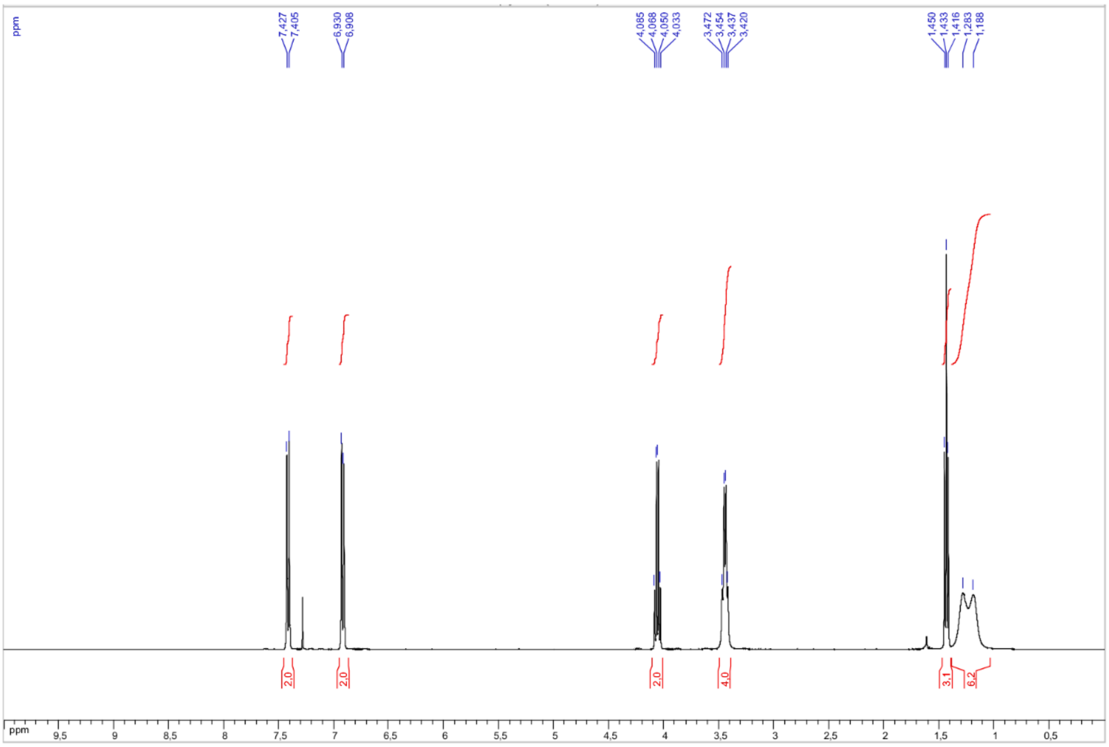

Figure S23. 6b ^{1}H NMR.

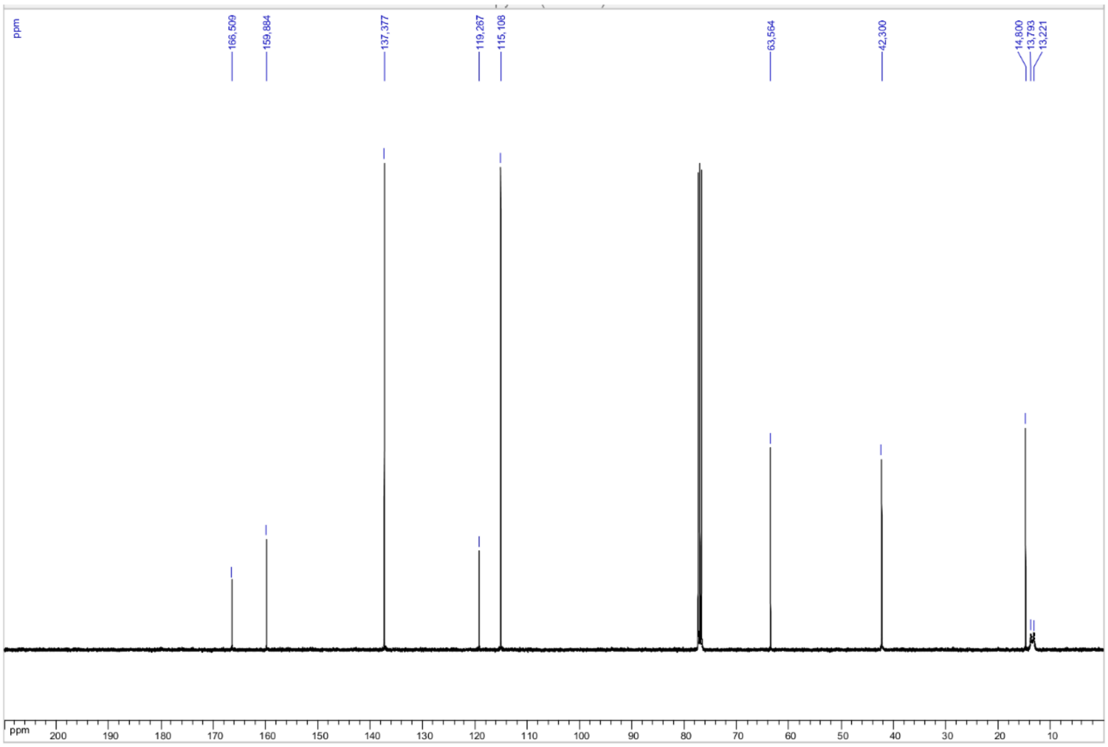

Figure S24. 6b ^{13}C NMR.

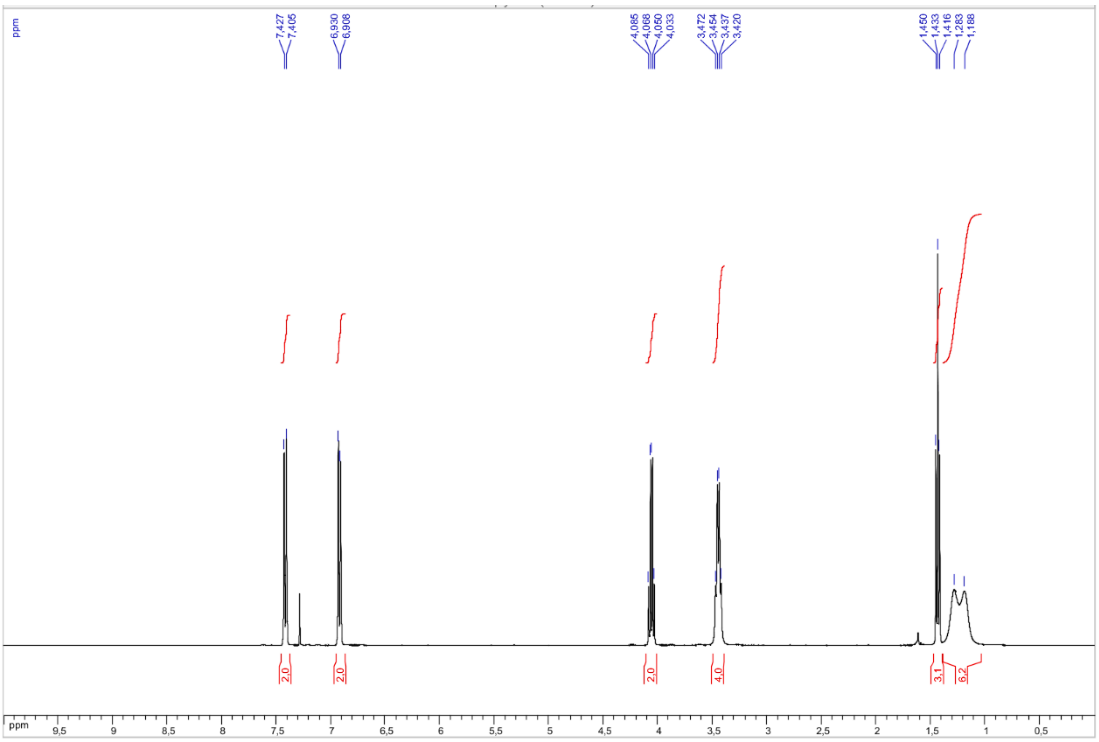

Figure S25. 6c ^{1}H NMR.

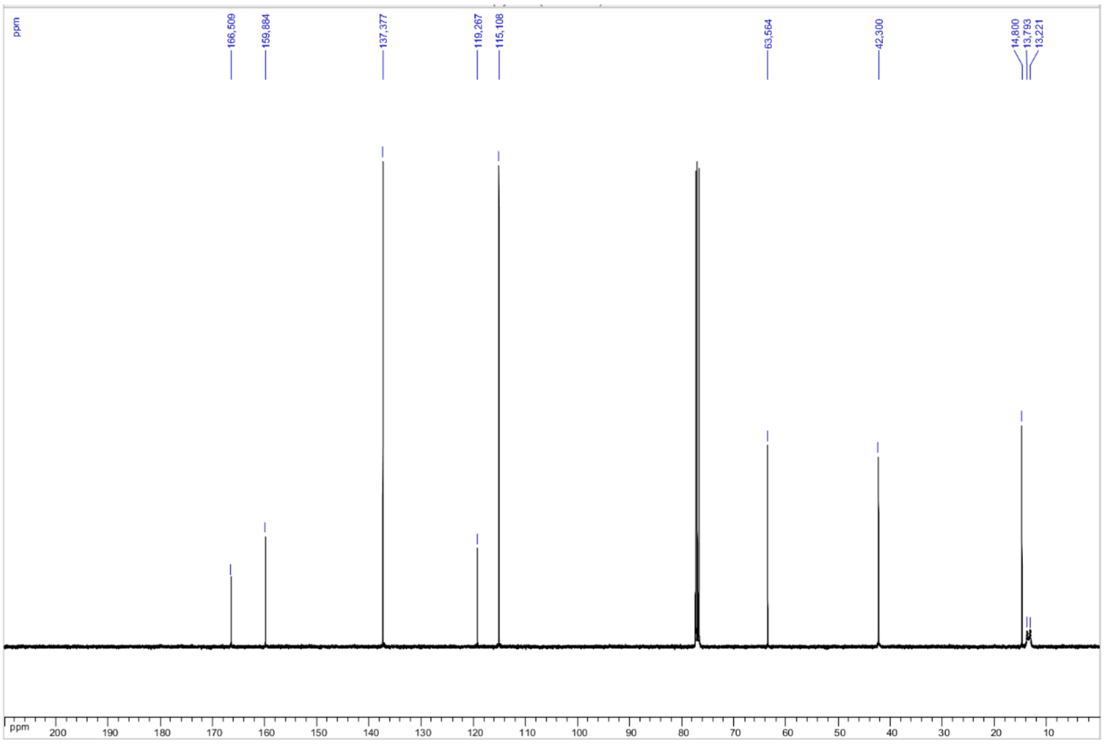

Figure S26. 6c ^{13}C NMR.

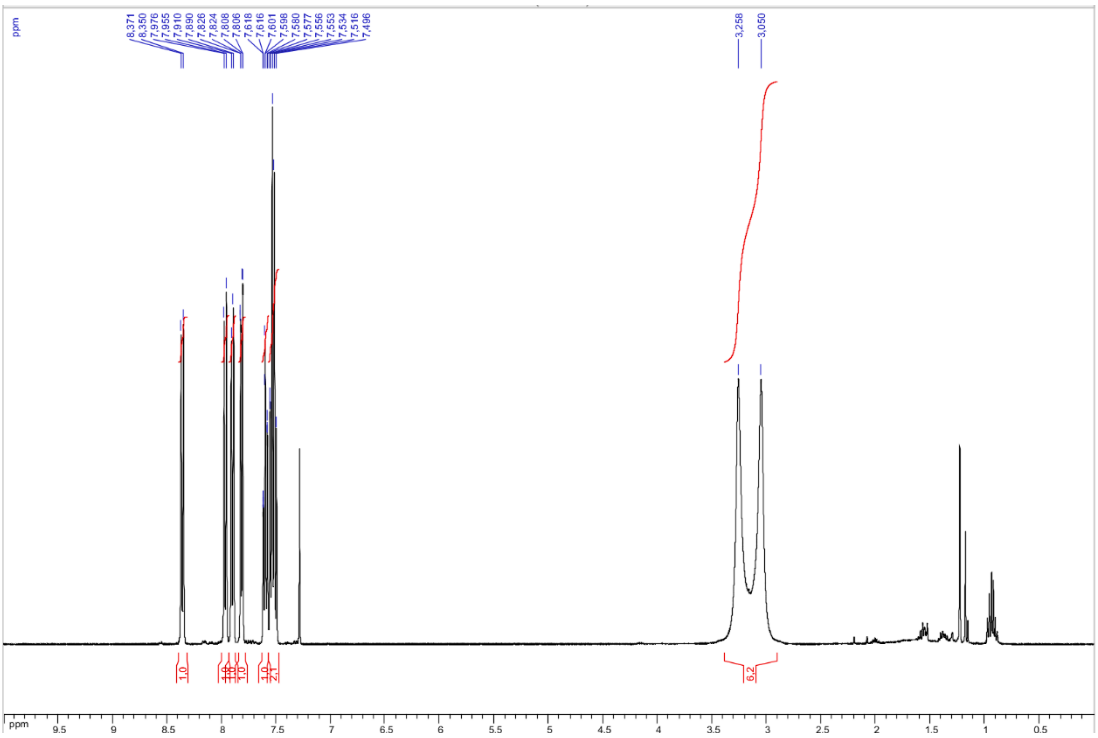

Figure S27. 6d ^{1}H NMR.

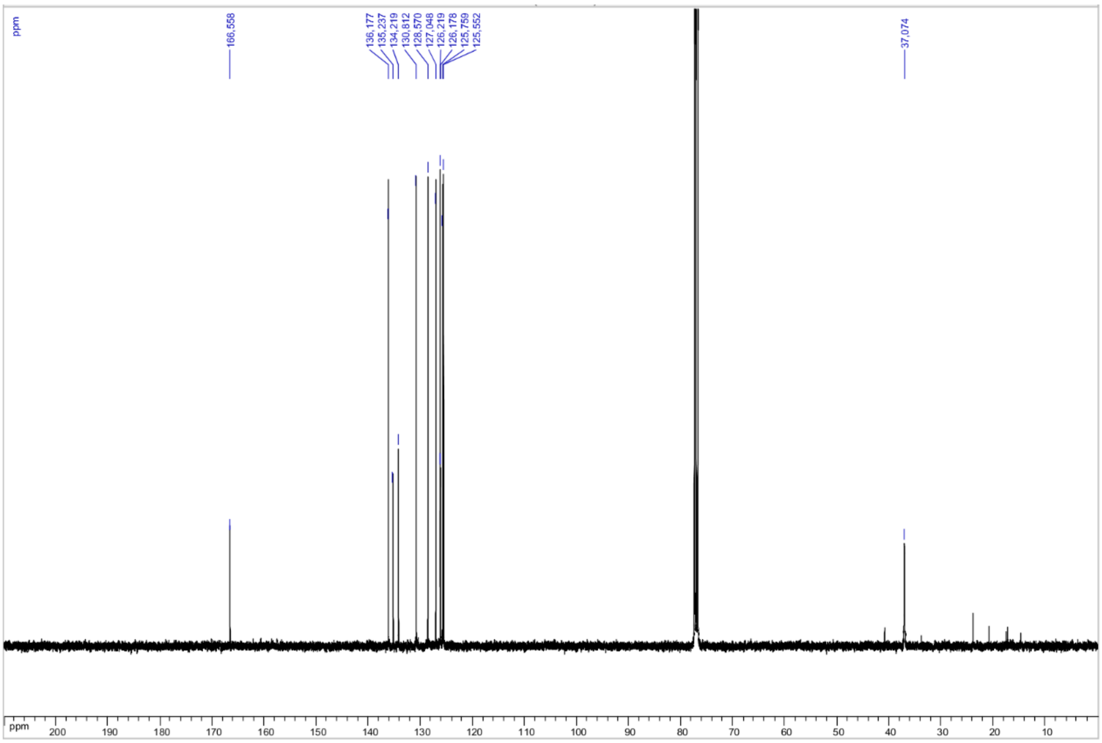

Figure S28. 6d ^{13}C NMR.

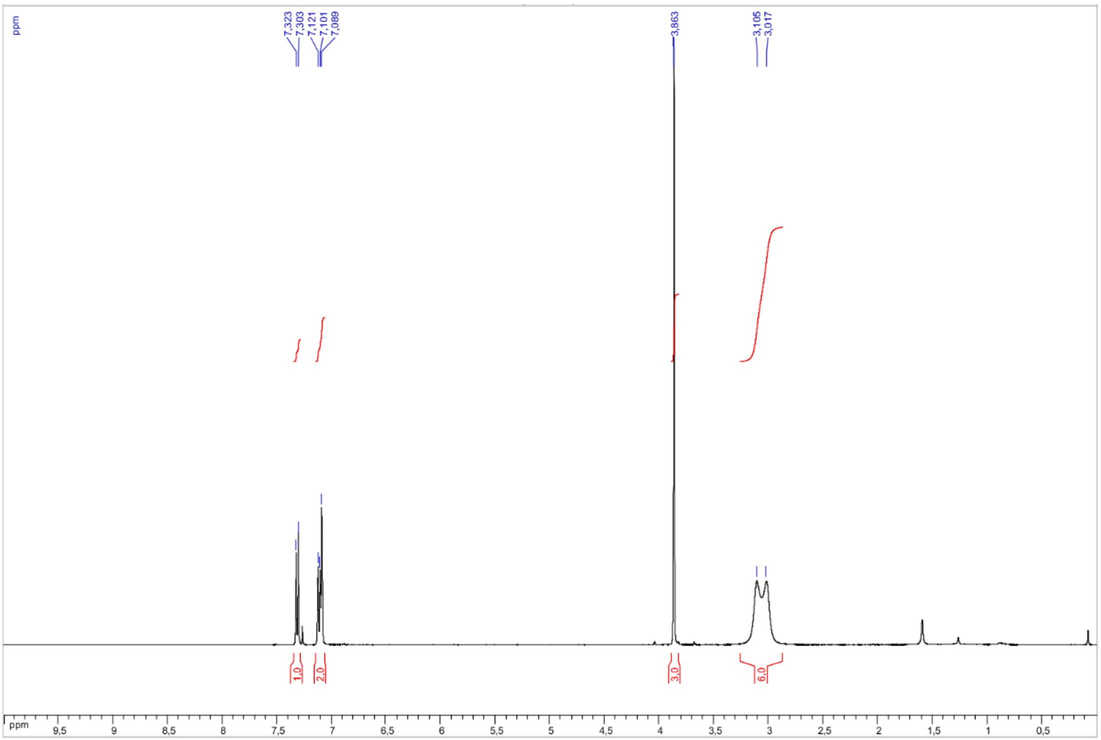

Figure S29. 6e ^{1}H NMR.

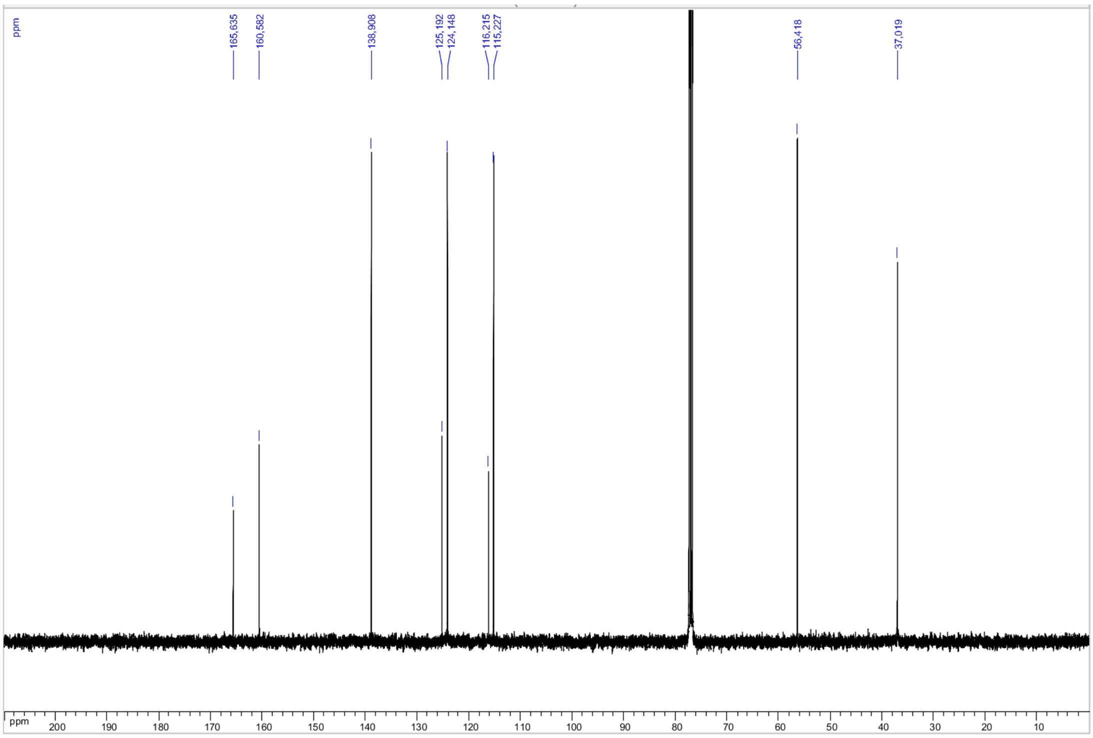

Figure S30. 6e ^{13}C NMR.

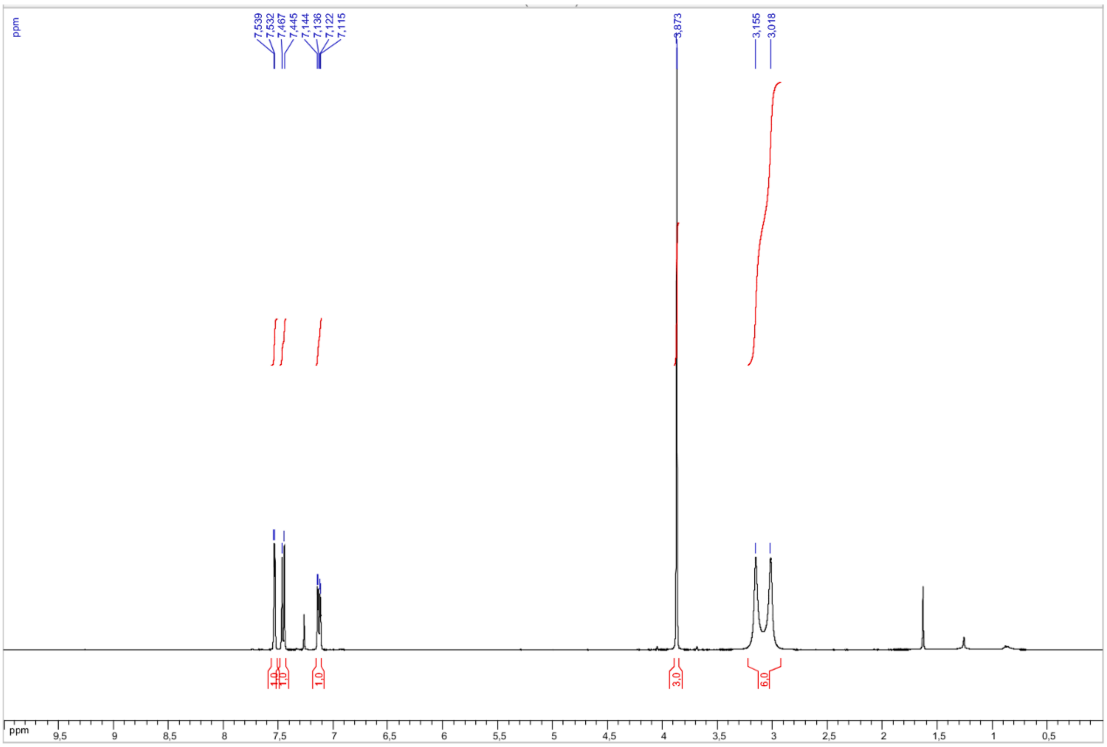

Figure S31. 6f ^{1}H NMR.

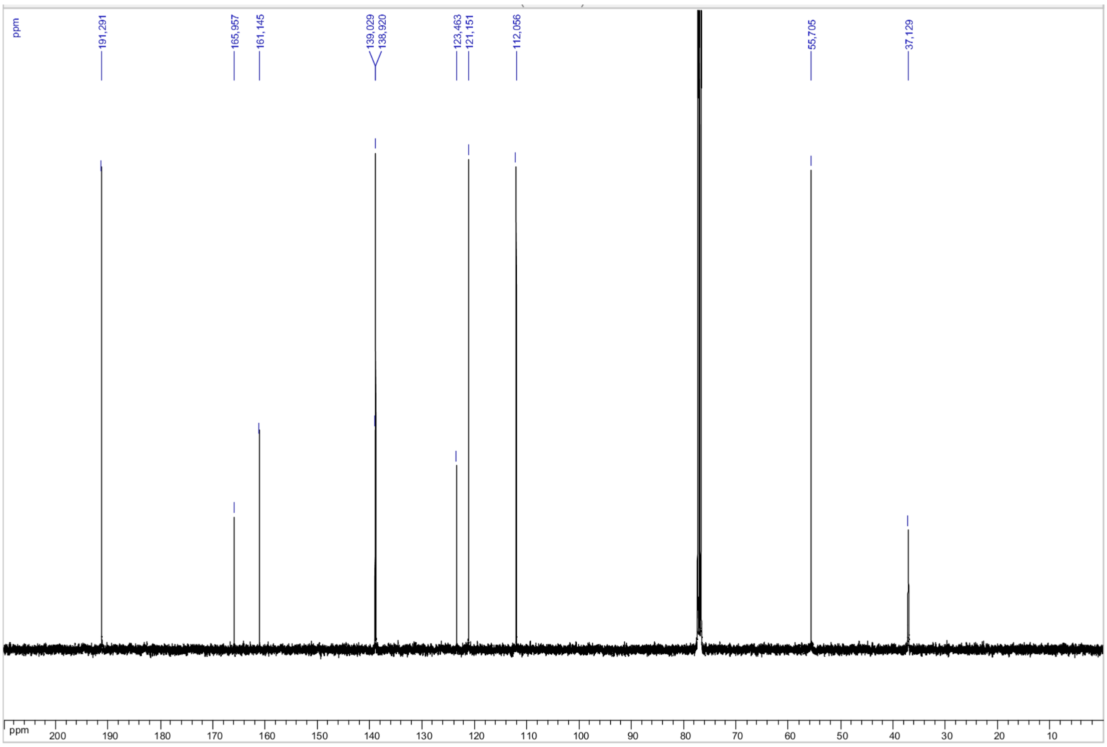

Figure S32. 6f ^{13}C NMR.

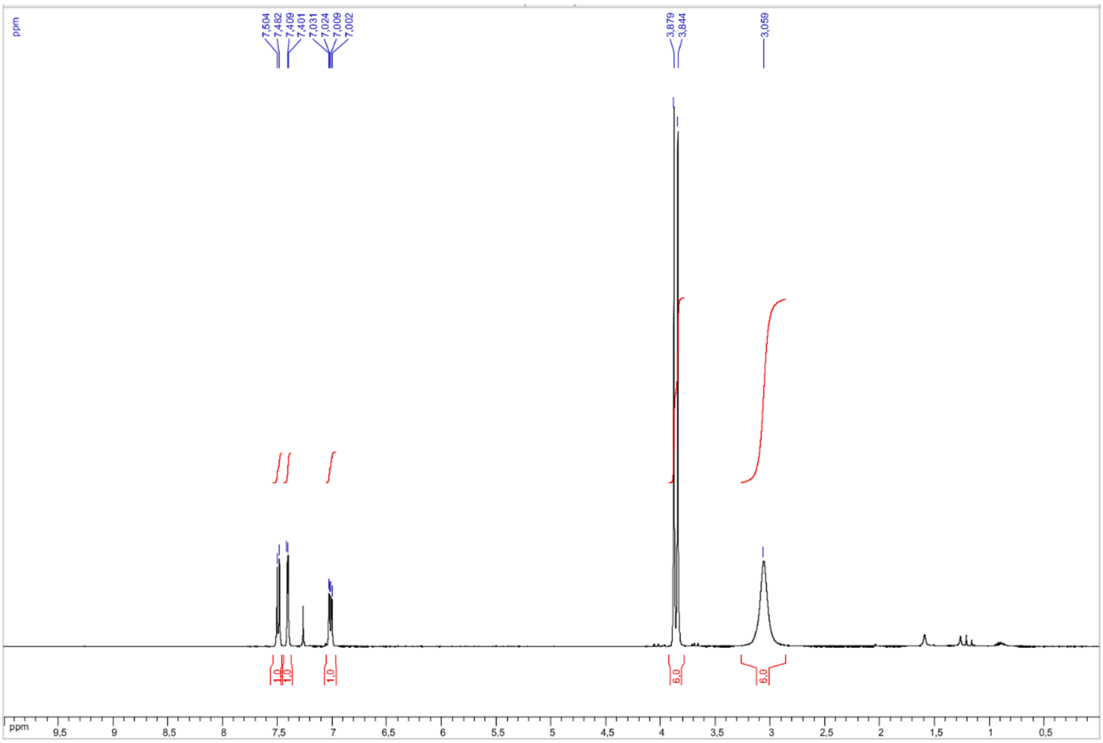

Figure S33. 6g ^{1}H NMR.

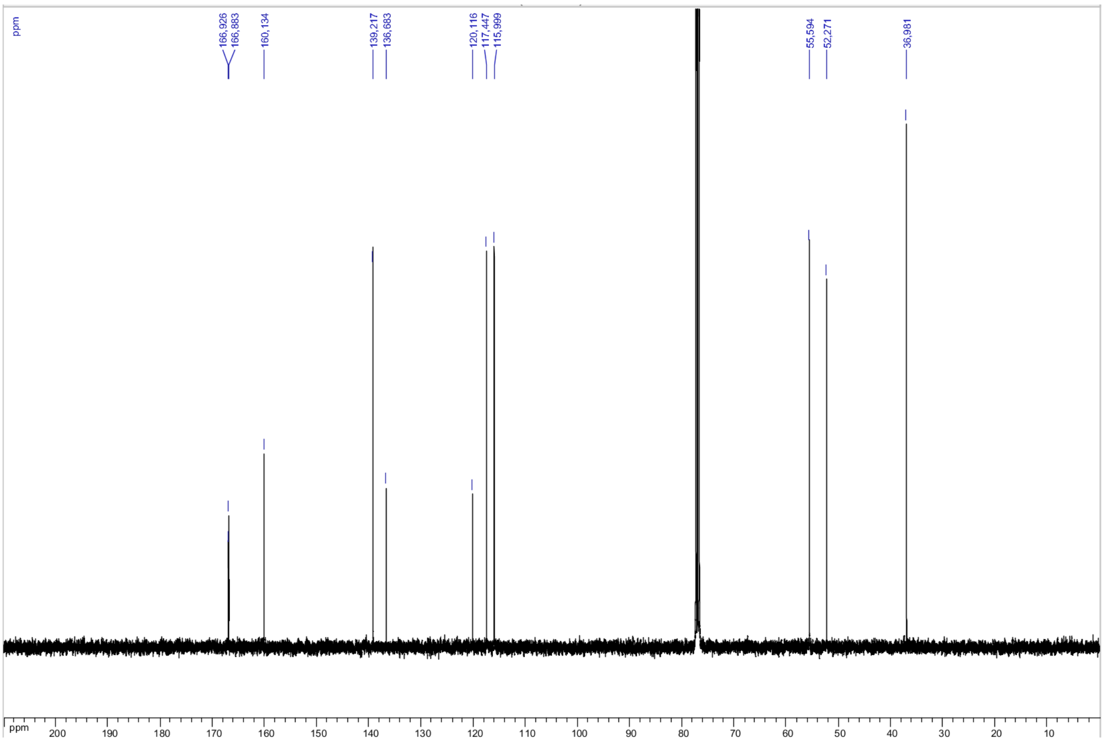

Figure S34. 6g ^{13}C NMR.

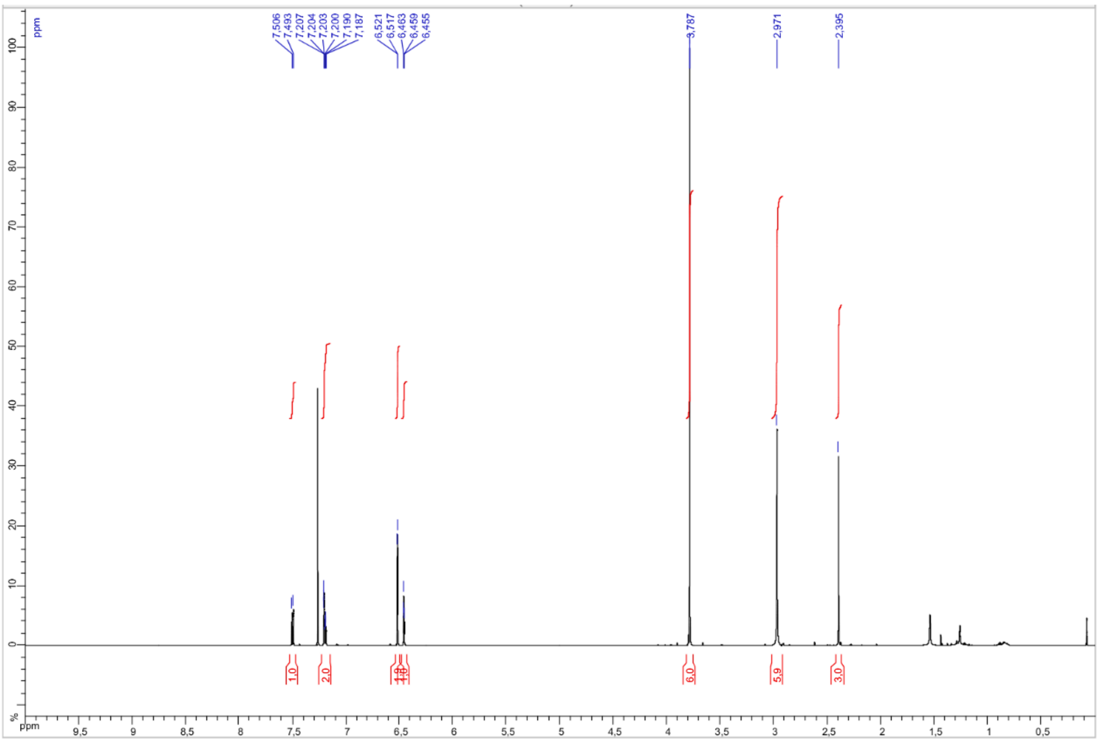

Figure S35. 6h ^{1}H NMR.

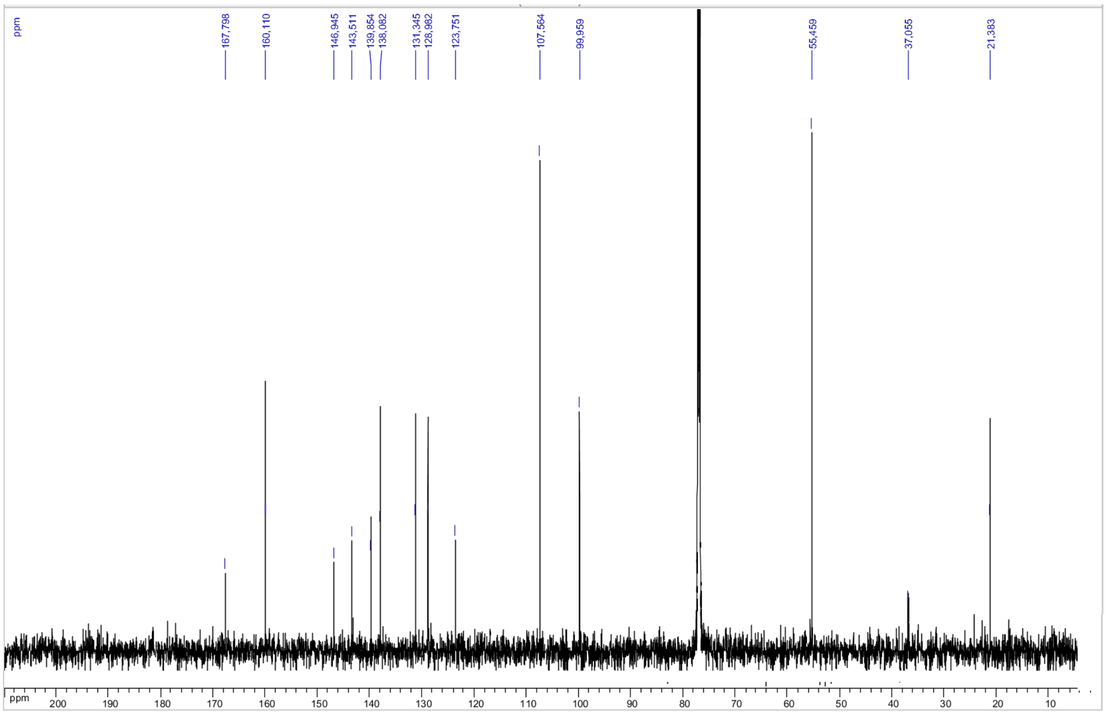

Figure S36. 6h ^{13}C NMR.
